# Systematically Exploring the Chemical Ingredients and Absorbed Constituents of *Polygonum capitatum* in Hyperuricemia Rat Plasma Using UHPLC-Q-Orbitrap HRMS

**DOI:** 10.3390/molecules27113521

**Published:** 2022-05-30

**Authors:** Huanyu Guan, Pengfei Li, Qian Wang, Fanli Zeng, Daoping Wang, Mei Zhou, Meng Zhou, Xun He, Shanggao Liao, Weidong Pan

**Affiliations:** 1 State Key Laboratory of Functions and Applications of Medicinal Plants & School of Pharmacy, Guizhou Medical University, Guiyang 550025, China; guanhuanyu@gmc.edu.cn (H.G.); wq112240920@163.com (Q.W.); fqzx2018chm@163.com (F.Z.); wangdaoping@gzcnp.cn (D.W.); mei_zhou0429@163.com (M.Z.); zhoumeng@gmc.edu.cn (M.Z.); hexun224@gmc.edu.cn (X.H.); 2 National Institute of Drug Clinical Trial, Guizhou Provincial People’s Hospital, Guiyang 550002, China; lipengfei@gz5055.com; 3 Key Laboratory of Chemistry for Natural Products of Guizhou Province and Chinese Academy of Sciences, Guiyang 550014, China

**Keywords:** *Polygonum capitatum*, UHPLC-Q-Orbitrap HRMS, chemical profiling, metabolites

## Abstract

*Polygonum capitatum* as an ethnic medicine has been used to treat urinary tract infections, pyelonephritis and urinary calculi. In our previous study, *P. capitatum* was found to have anti-hyperuricemia effects. Nevertheless, the active constituents of *P. capitatum* for treating hyperuricemia were still unclear. In this study, an ultra-high-performance liquid chromatography coupled to quadrupole/orbitrap high-resolution mass spectrometry (UHPLC-Q-Orbitrap HRMS) was used to comprehensively detect the chemical ingredients of *P. capitatum* and its absorbed constituents in the plasma of hyperuricemia rats for the first time. Xcalibur 3.0 and Compound Discoverer 2.0 software coupled to mzCloud and ChemSpider databases were utilized for qualitative analysis. A total of 114 chemical components including phenolics, flavonoids, tannins, phenylpropanoids, amino acids, amides and others were identified or tentatively characterized based on the exact mass, retention time and structural information. Compared to the previous *P. capitatum* study, an additional 66 different components were detected. Moreover, 68 related xenobiotics including 16 prototype components and 52 metabolites were found in the plasma of hyperuricemia rats. The metabolic pathways included ring fission, hydrolysis, decarboxylation, dehydroxylation, methylation, glucuronidation and sulfation. This work may provide important information for further investigation on the active constituents of *P. capitatum* and their action mechanisms for anti-hyperuricemia effects.

## 1. Introduction

Hyperuricemia (HUA) is one of the most common metabolic conditions characterized by abnormally increased serum urate levels. Long-term HUA is a main etiologic factor for the deposition of monosodium urate crystals (MSU) in joints and soft tissues resulting in gout [1]. Moreover, HUA is associated with incidences of hypertension, diabetes, obesity and chronic kidney disease [2]. Allopurinol, febuxostat and benzbromarone were selected as anti-hyperuricemic agents, although these agents exhibited some adverse effects including hypersensitivity, cardiovascular mortality risk and hepatic toxicity [3,4,5].

Traditional Chinese medicine (TCM) and ethnic medicine have been applied to treat hyperuricemia and gout for over thousands of years with their own unique advantages. TCM and ethnic medicine were considered important resources for discovering multitarget drugs for the treatment of hyperuricemia and gout. *Polygonum capitatum* Buch.-Ham. ex D. Don, named Touhualiao in Chinese, was utilized as Miao ethnic medicine in China to treat urinary tract infections, pyelonephritis and urinary calculi [6]. In our previous study, *P. capitatum* was found to reduce serum urate levels to treat hyperuricemia and gouty arthritis without renal toxicities. The underlying action mechanism of *P. capitatum* involved inhibiting the expression and function of xanthine oxidase and decreasing the expressions of glucose transporter 9 (GLUT9) and urate transporter 1 (URAT1) [7]. Despite the remarkable efficacy of *P. capitatum* for anti-hyperuricemia and anti-gouty arthritis, the active constituents of *P. capitatum* related to the pharmacological effect are still not clear.

A range of the active constituents of TCM and ethnic medicine is an essential prerequisite for executing pharmacological effects. Profiling the chemical ingredients, the absorbed constituents and metabolites is beneficial for elucidating the pharmacological materials of TCM and ethnic medicine. Traditional separation technologies have been used to isolate and obtain pure components from *P. capitatum* including triterpenes [8], phenolics [8,9,10], flavonoids [8,9,10], lignans [8,9], alkaloids [10] and tannins [11], although the technology was time-consuming and it was often difficult to acquire the substances at low concentrations. Only partial phenolics of *P. capitatum* were identified by ultra-high-performance liquid chromatography-photodiode array detection coupled with triple quadrupole mass spectrometry (UHPLC-PDA-QqQ-MS) [12] and UHPLC with time-of-flight mass spectrometry (UHPLC-TOF-MS) [13]. These were not sufficient for the research on the active constituents and action mechanisms of *P. capitatum* for anti-hyperuricemia and anti-gouty arthritis. Therefore, it is necessary to comprehensively identify and characterize the chemical constituents of *P. capitatum* and its absorbed components in hyperuricemia rats.

UHPLC coupled with quadrupole/orbitrap high-resolution mass spectrometry (UHPLC-Q-Orbitrap HRMS) technology provided a sensitive and high-resolution platform for the analysis of chemical constituents at μg/kg concentration levels in complex matrix samples. The data-dependent acquisition mode of Q-Orbitrap HRMS provides MS/MS spectra with accurate mass data [14,15] which are beneficial for identifying and characterizing unknown compounds in TCM and ethnic medicine. Q-Orbitrap HRMS was also a powerful analytical technology for elucidating the metabolism of TCM and ethnic medicine in vivo due to its high sensitivity, high resolution and fast scanning capability.

In this study, a UHPLC-Q-Orbitrap HRMS method was employed to systematically clarify the chemical constituents of *P. capitatum* for the first time. Additionally, the prototypes and metabolites of *P. capitatum* in hyperuricemia rat plasma were also analyzed by the UHPLC-Q-Orbitrap HRMS technology based on neutral loss and metabolism types of representative components. Ultimately, 114 chemical constituents were tentatively identified or characterized from *P. capitatum*. Compared to the previous *P. capitatum* study using LC-MS, additional 66 different components were detected in this study. Among these, two new compounds were found, and 7 compounds were discovered in *P. capitatum* for the first time. A total of 68 related xenobiotics including 16 prototypes and 52 metabolites were detected in the hyperuricemia rats. Among them, 14 prototypes and 50 metabolites were reported for the first time. This study helped illustrate the active components and action mechanisms of *P. capitatum* for anti-hyperuricemia and anti-gouty arthritis.

## 2. Results and Discussion

### 2.1. UHPLC-Q-Orbitrap HRMS Analysis of P. capitatum Extract

**Figure 1:**
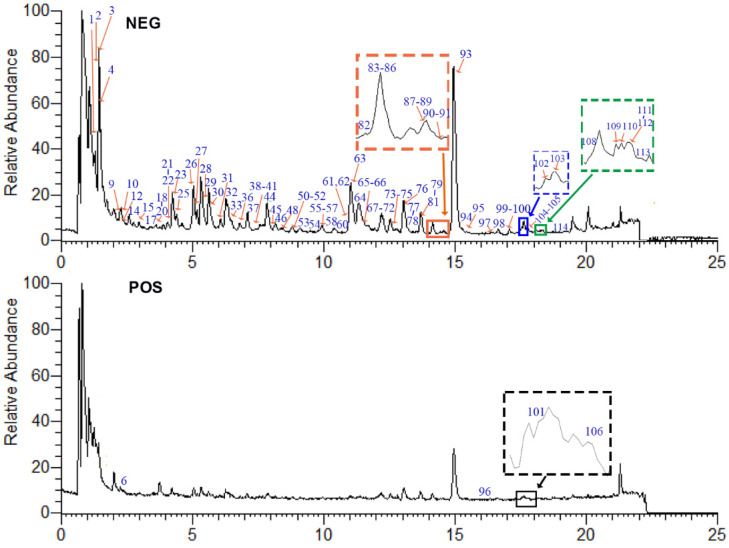
The TIC chromatograms of *P. capitatum* extract in negative (NEG) and positive (POS) ion modes.

UHPLC-Q-Orbitrap HRMS method was employed to profile the chemical constituents in *P. capitatum* extract. Under the optimized UHPLC-Q-Orbitrap HRMS conditions, the total ion current (TIC) chromatograms of *P. capitatum* extract in negative and positive ion modes are shown in Figure 1. The elemental compositions for the compound and the fragment ion were predicted within a mass tolerance of ±5 ppm. The chemical structures of the components in *P. capitatum* were elucidated by comparing their retention time, exact mass and structural information with those of authentic standards or available literature data. As a result, a total of 114 components from *P. capitatum* were unambiguously identified or tentatively characterized (Table S1 in Supplementary Materials), including 30 phenolic acids, 38 flavonoids, 16 phenylpropanoids, 10 tannins, 10 phenolics, 3 amino acids, 3 amides and 4 others. The chemical structures of the detected constituents were shown in Figure S1 in Supplementary Materials. Among these compounds, compounds **59**, **77**, **84**, **96**, **97**, **110** and **112** were found in *P. capitatum* for the first time. Although positive and negative ion modes were employed, more peak signals and higher sensitivities were obtained in the negative ion mode. The fragment ions in the negative and positive ion modes are listed in Table S1.

#### 2.1.1. Phenolic Acids

The mass signals for phenolic acids in negative ion mode were observed. The quasi-molecular ions of phenolic acids preferred to produce the corresponding product ions by neutral loss of H_2_O, CO and CO_2_. Compounds **4** and **14** were unambiguously identified as gallic acid and protocatechuic acid, respectively, by comparing their retention time and mass data with those of reference standards. The [M − H]^−^ of gallic acid at *m/z* 169.0133 lost a unit of CO_2_ to form the product ion at *m/z* 125.0233 and subsequently discarded a H_2_O unit and a CO group to yield the ions at *m/z* 107.0126 and 97.0283. The [M − H]^−^ ion of compound **55** was 14.0158 Da (CH_2_) more massive than that of gallic acid. The ions at *m/z* 168.0055 and 165.0187 were formed from the [M − H]^−^ ion through losing ·CH_3_ and H_2_O. Moreover, the prominent ion at *m/z* 139.0390 was assigned as [M − H − CO_2_]^−^. Compound **55** was tentatively identified as the reported 4-*O*-methylgallic acid [12]. Similarly, compounds **50** and **52** gave the [M − H]^−^ ions at *m/z* 197.0448 and 197.0449 with the same predicted molecular formulae (C_9_H_9_O_5_^−^), which was C_2_H_4_ moiety more massive than that of gallic acid. Notably, the product ions at *m/z* 169.0133 of compound **50** corresponding to [M − H − C_2_H_4_]^−^ indicated the existence of ethyl moiety. The fragment ion at *m/z* 125.0233 ([M − H − C_2_H_4_ − CO_2_]^−^) was also observed in the tandem mass spectrometry (MS^2^) of compound **50**. Therefore, compound **50** was tentatively identified as the reported ethyl gallate [16]. Different from the fragmentation pattern of compound **50**, compound **52** generated the product ions at *m/z* 182.0212 ([M − H − CH_3_·]^−^) and 166.9976 ([M − H − 2 × CH_3_·]^−^) suggesting the presence of two methyl groups in compound **52**. Compound **52** was assigned as syringic acid [17]. Compounds **17** and **35** eluted at 3.20 min and 7.07 min showed additional hexoside groups (162 Da) compared to compounds **52** and **50**. The fragmentation pathways of the aglycone ions were similar to those of compounds **52** and **50**. Compounds **17** and **35** were tentatively identified as syringic acid-*O*-hexoside and ethyl gallate-*O*-hexoside. Compound **13** gave a [M − H]^−^ ion at *m/z* 329.0858 and exhibited the fragment ions at *m/z* 167.0341 ([M − H − hexose sugar]^−^), 152.0105 ([M − H − hexose sugar − CH_3_·]^−^), 123.0440 ([M − H − hexose sugar − CO_2_]^−^) and 108.0205 ([M − H − hexose sugar-CO_2_ − CH_3_·]^−^) assigned as the moiety of vanillic acid. Based on the above observation, compound **13** was tentatively identified as vanillic acid-*O*-hexoside. Compound **34** exhibited the quasi-molecular ion at *m/z* 481.0985 ([M − H]^−^) which was 152.0127 Da (C_7_H_4_O_4_) more massive than that of compound **13**. The [M − H]^−^ ion of compound **34** lost a vanillic acid residue and a dehydrated hexose moiety in succession to form the ions at *m/z* 313.0564 and 169.0133. Notably, the characteristic fragment ions at *m/z* 169.0133, 125.0232 and 97.0282 assigned as gallic acid were observed in the MS^2^ spectrum of compound **34**, which primarily implied a galloyl residue connected to the vanillic acid-*O*-hexoside. Due to the existences of vanillic acid [17] and 2-methoxy-4-hydroxyphenol-1-*O-β*-d-(6′-*O*-galloyl) glucopyranoside [10] in *P. capitatum*, compound **34** was tentatively identified as vanillic acid-4-*O*-(6′-*O*-galloyl)-glucopyranoside.

#### 2.1.2. Flavonoids

Flavonoids refer to a class of natural compounds possessing a chemical skeleton of C6-C3-C6. A total of 38 flavonoids were detected in *P. capitatum*, including flavonols, flavan-3-ols and flavanones. Compounds **26**, **67**, **69**, **75**, **76**, **93**, **105** and **107** were confirmed as (+)-catechin, myricetrin, quercetin-3-*O*-(2″-*O*-galloyl)-*β*-d-glucopyranoside, rutin, quercetin-3-*O-β*-d-glucopyranoside, quercitrin, quercetin and 3″-*O*-galloylquercitrin compared by authentic reference standards.

**Figure 2:**
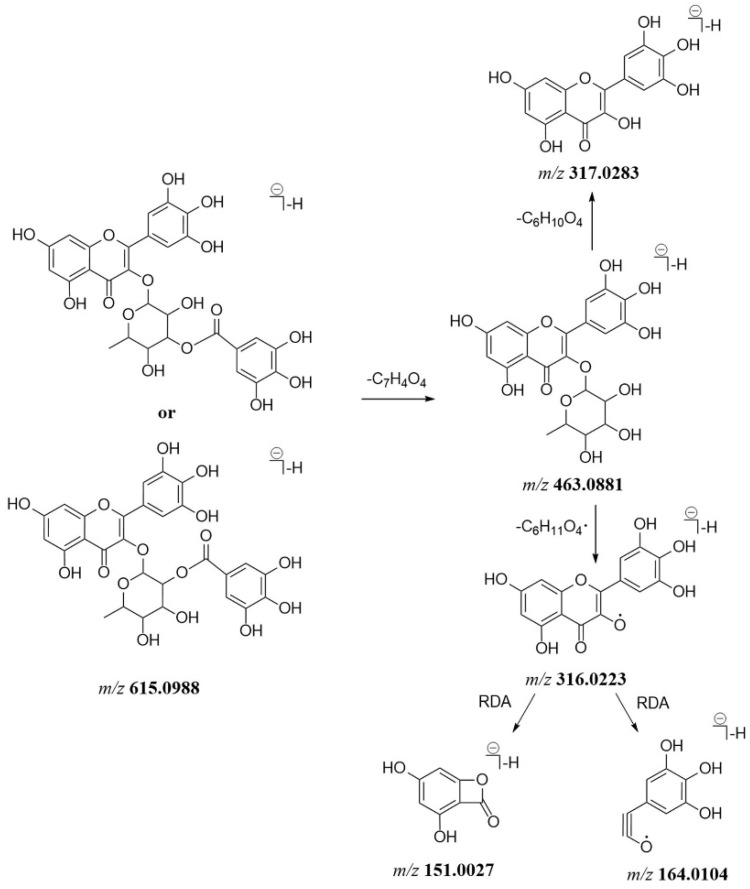
The proposed mass fragmentation pathway of compound **97**.

Generally, flavonoid *O*-glycosides usually lose saccharide moieties to produce the corresponding aglycone ions. Most of the flavonoid *O*-glycosides in *P. capitatum* were characterized by galloyl groups attached to monosaccharide residues. In the MS^2^ spectra, the loss of a galloyl moiety (152 Da) was the characteristic fragmentation pattern of flavonoid *O*-glycosides in *P. capitatum.* For flavonoid aglycones, retro-diels-alder (RDA) fragmentation reaction and neutral losses of H_2_O (18 Da), CO (28 Da) were involved. As the isomers of quercetin-3-*O*-(2″-*O*-galloyl)-*β*-d-glucopyranoside (compound **69**), compounds **79** and **97** showed the [M − H]^−^ ions at *m/z* 615.0987 (err. 1.02 ppm) and 615.0988 (err. 1.23 ppm) with the same predicted molecular formulae (C_28_H_23_O_16_^−^). Compound **79** gave a similar fragment pattern to that of compound **69**, where the [M − H]^−^ ion continuously lost a galloyl unit and a hexose residue to form the ion at *m/z* 463.0886 and 301.0352. The fragment ions at *m/z* 151.0027 and 107.0126 were generated from the aglycone ion through the RDA fragmentation reaction. Compound **79** was tentatively identified as the reported quercetin-3-*O*-(3″-*O*-galloyl)-*β*-d-glucopyranoside [18]. The MS^2^ spectrum for compound **97** showed the product ions at *m/z* 463.0881 and 316.0223 resulting from the loss of a galloyl unit and a rhamnosyl residue in succession. The fragment ions observed at *m/z* 164.0104 and 151.0027 were assigned as the [^1,3^A]^−^ and [^1,3^B]^−^ products of the RDA fragmentation pathway. The observed ions at *m/z* 463.0881, 316.0223, 164.0104 and 151.0027 were characteristic of myricetin-*O*-rhamnoside. Myricetin-3-*O*-rhamnoside-gallate has been identified as a main chemical constituent of *Polygonum neofiliforme* as homologous plant of *P. capitatum* [19]. Moreover, the compounds quercetin-3-*O*-(2″-*O*-galloyl)-rhamnopyranoside and quercetin-3-*O*-(3″-*O*-galloyl)-rhamnopyranoside were present in *P. capitatum* [18]. Compound **97** was tentatively identified as myricetin-3-*O*-(2″-*O*-galloyl)-rhamnopyranoside or myricetin-3-*O*-(3″-*O*-galloyl)-rhamnopyranoside. The proposed mass fragmentation pathway of compound **97** is shown in Figure 2.

Compound **87** detected at 14.50 min showed a quasi-molecular ion at *m/z* 629.0785 (C_28_H_21_O_17_^−^) and lost a galloyl moiety to generate the product ions at *m/z* 477.0678. The above [M − H]^−^ ion and product ions of compound **87** were 13.9797 Da higher than the corresponding ions of compound **79**. The observed ions at *m/z* 327.0357 and 175.0240 of compound **87** suggested a glucuronic acid moiety was connected to the galloyl group. Furthermore, the ions at *m/z* 301.0353, 151.0027 and 107.0126 were assigned as quercetin. Therefore, compound **87** was tentatively identified as a new compound, quercetin 3-*O*-galloylglucuronide. Similarly, compounds **102** and **104** were tentatively identified as the reported quercetin-3-*O*-(6″-*O*-trans-feruloyl)-*β*-d-galactopyranoside [20] and quercetin-3-*O*-(4″-*O*-acetyl)-*α*-l-rhamnopyranoside [21], respectively. Compound **49** eluted at 8.63 min exhibited an [M − H]^−^ ion at *m/z* 735.1563 and gave the product ions at *m/z* 583.1091 and 447.0930 through losing a protocatechuoyl moiety and a galloyl group in succession, suggesting the existence of protocatechuoyl and galloyl residues. In addition, the characteristic ions at *m/z* 447.0930, 301.0348 and 243.0295 in the MS^2^ spectra implied the presence of quercetin-*O*-rhamnoside. Although the positions of the protocatechuoyl moiety and the galloyl group were unclear, the finding of quercetin-3-*O*-(2″-*O*-protocatechuoyl)-rhamnoside and 3″-*O*-galloylquercitrin in this plant implied the protocatechuoyl and galloyl moieties were connected to the monosaccharide residue of compound **49**. Therefore, compound **49** was tentatively identified as a new compound, quercetin-3-*O*-(protocatechuoyl-galloyl)-rhamnoside.

#### 2.1.3. Phenylpropanoids

**Figure 3:**
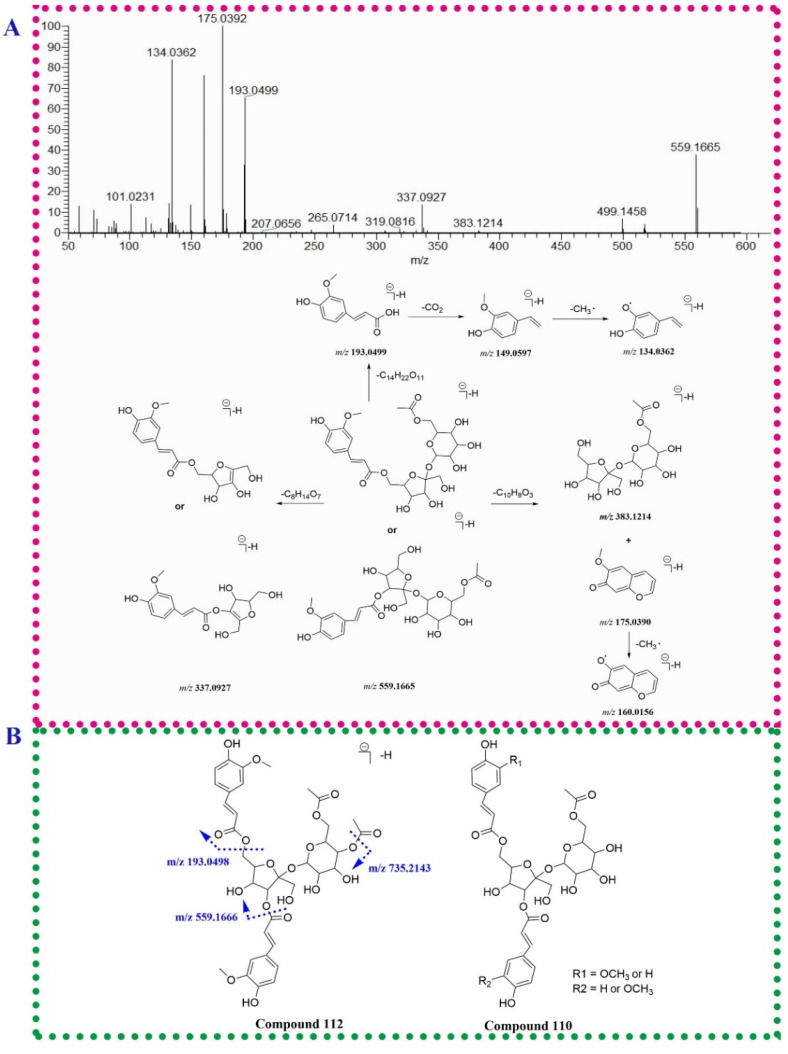

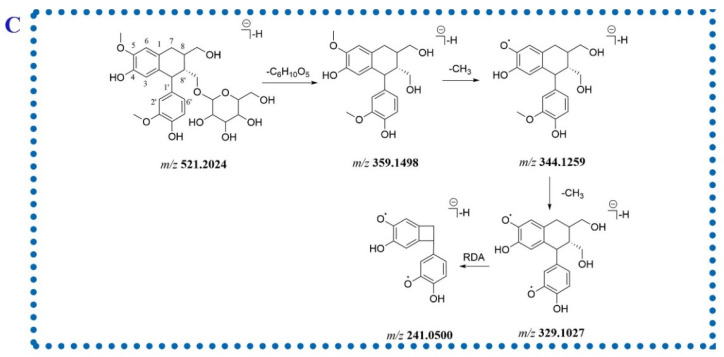
The fragmentation patterns of compounds **84** (**A**), **110** (**B**) and **77** (**C**).

Phenylpropanoids in *P. capitatum* were divided into phenylpropanoid sucrose esters, lignans and chromones derivatives. Compound **111** (t_R_ = 18.36 min) produced an [M − H]^−^ ion at *m/z* 735.2141 (C_34_H_39_O_18_^−^, err. 1.31). In the MS^2^ spectrum, the fragment ions at *m/z* 559.1661 (C_24_H_31_O_15_^−^) and 175.0392 (C_10_H_7_O_3_^−^) were produced via the loss of the dehydrated feruloyl group. The ion at *m/z* 559.1661 lost an acetylated hexosyl residue to generate the ion at *m/z* 337.0931, and subsequently discarded another hexosyl residue to form a feruloyl moiety (*m/z* 193.0498, C_10_H_9_O_4_^−^). Compound **111** was tentatively identified as the reported bistoroside B [22]. Compared to compound **111**, compound **84** (t_R_ = 14.16 min, *m/z* 559.1665, [M − H]^−^, C_24_H_31_O_15_^−^) exhibited the absence of a feruloyl residue and showed a similar mass fragmentation pathway in compound **111**. Compound **84** was tentatively identified as 6′-acetyl-6(or 3)-feruloylsucrose. This compound was firstly found in the *Polygonum* genus. The mass data and the fragmentation pathway of 6′-acetyl-6(or 3)-feruloylsucrose was proposed for the first time in this study (Figure 3A). Similarly, compound **112** (t_R_ = 18.36 min, *m/z* 777.2244, [M − H]^−^, C_36_H_41_O_19_^−^) showed an additional acetyl moiety (42.0103 Da) compared to compound **111**. Based on the reported mass data [23], compoumd **112** was tentatively identified as smilaside A. Compound **110** possessed an [M − H]^−^ ion at *m/z* 705.2040 (C_33_H_37_O_17_^−^) and gave the product ions at *m/z* 559.1662 (C_24_H_31_O_15_^−^), 163.0389 (C_9_H_7_O_3_^−^) and 145.0284 (C_9_H_5_O_2_^−^), suggesting the existence of a coumaroyl moiety. The characteristic ions of 6′-acetyl-6(or 3)-feruloylsucrose at *m/z* 559.1662, 337.0943, 193.0499 and 175.0392 were also observed in the MS^2^ spectrum of compound **110**. Compound **110** was tentatively identified as 6′-acetyl-3(or 6)-feruloyl-6(or 3)-coumaroylsucrose (Figure 3B).

Two isomeric compounds, **77** and **90**, were eluted at 13.20 and 14.83 min possessing identical molecular formulae (C_26_H_33_O_11_^−^). For compound **77**, the fragment ions at *m/z* 359.1498, 344.1259 and 329.1027 in the MS^2^ spectrum were generated via losing a hexosyl moiety and subsequent discarding two methyl groups. The RDA fragment ion at *m/z* 241.0500 was corresponding to the cleavage of 8-8′ bond (Figure 3C). Due to the presence of isolariciresinol-9′-*O*-*β*-d-xylopyranoside in *P. capitatum*, compound **77** was tentatively identified as isolariciresinol-9′-*O*-glucopyranoside. Compound **90** neutrally lost a pentose residue (132 Da) to form the aglycone ion at *m/z* 389.1605 and lost three methyl groups in succession to generate the ions at *m/z* 374.1370, 359.1136 and 344.0892, respectively, implying the existence of three methoxy groups in the aglycone moiety. Therefore, compound **90** was tentatively identified as 3(or 5′)-methoxyisolariciresinol-9′-*O*-xylopyranoside.

#### 2.1.4. Tannins

Tannins identified in *P. capitatum* in this study were classified into proanthocyanidins and ellagitannins. Proanthocyanidins were condensed tannins composed of oligomers and polymers of flavan-3-ol moieties linked mainly through 4-8′ bonds. RDA reaction, heterocyclic ring fission (HRF) and quinone methide (QM) cleavage were the main mass fragmentation patterns of proanthocyanidins [24]. Ellagitannins belonging to hydrolyzable tannins consisted of hexahydroxydiphenoyl (HHDP) groups and related acyl groups. The characteristic fragment ion at *m/z* 300.9991 (C_14_H_5_O_8_^−^) in the MS^2^ spectrum of ellagitannin was corresponding to an ellagic acid moiety.

**Figure 4:**
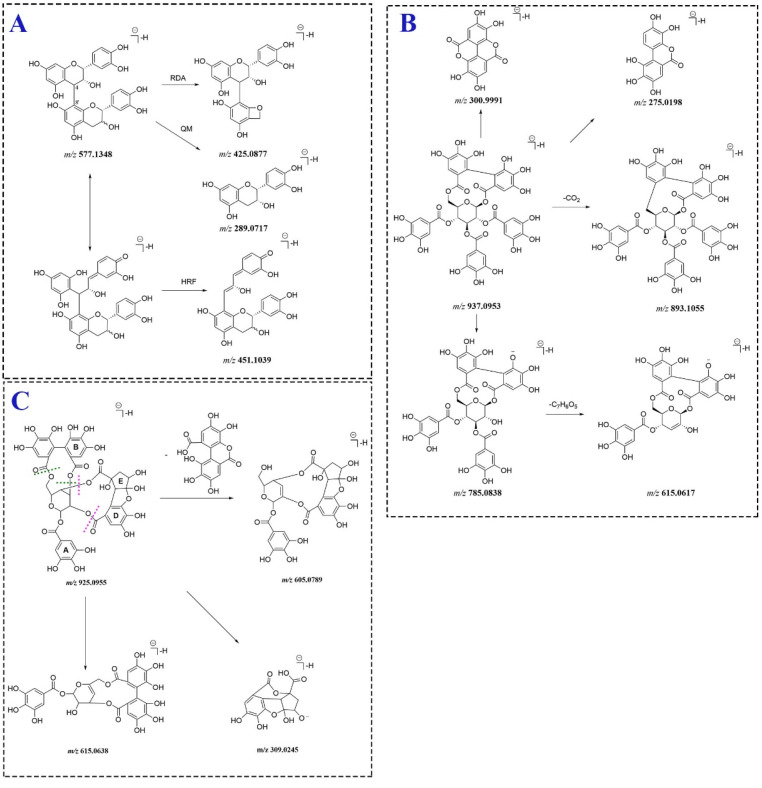
The fragmentation pathways of compounds **25** or **32** (**A**), **64** (**B**) and **33** (**C**). The dotted purple and green lines represent the cleavage positions of compound **33** in MS^2^ spectrum.

A pair of isomer compounds **25** and **32** both showed the deprotonated ions at *m/z* 577.1348 ([M − H]^−^, C_30_H_25_O_12_^−^) and the fragment ions at *m/z* 425.0877, 451.1039 and 289.0717 generated through RDA fragment reaction, HRF fragmentation and QM cleavage, respectively (Figure 4A), which suggested that compounds **25** and **32** were the reported procyanidin B1/B2 [25]. Compounds **43**, **51** and **53** all produced the deprotonated ions at *m/z* 729.1458 (C_37_H_29_O_16_^−^) and lost a galloyl group to generate the fragment ions at *m/z* 577.1352 (C_30_H_25_O_12_^−^). The subsequent fragmentation pathways of the ion at *m/z* 577.1352 were similar to those of compounds **25** and **32**. Compounds **43**, **51** and **53** exhibited an additional galloyl residue compared to compounds **25** and **32**. Since catechin-3-*O*-gallate had been found in *P. capitatum,* compounds **43**, **51** and **53** were tentatively identified as 3(or 3′)-*O*-galloyl(epi)catechin-(4,8′)-(epi)catechin.

Compound **64** (t_R_ = 11.36 min) had a [M − H]^−^ ion at *m/z* 937.0953 (C_41_H_29_O_26_^−^) and gave the fragment ions at 893.1055 and 785.0838 generated from the loss of a CO_2_ unit and a galloyl residue, respectively. The product ion at *m/z* 785.0838 continuously discarded a galloyl residue to form the ion at *m/z* 615.0617. In addition, the ions at *m/z* 300.9991and 275.0198 were assigned to the moieties of ellagic acid and urolithin M5. Compound **64** was tentatively identified as davidiin. The proposed mass fragment pathway of compound **64** was shown in Figure 4B. Compound **33** (t_R_ = 6.71 min) exhibited an [M − H]^−^ ion at *m/z* 925.0955 (C_40_H_29_O_26_^−^). The MS^2^ spectra for compound **33** revealed the intense product ion at *m/z* 605.0789 resulting from the neutral loss of a dehydrated HHDP residue (C_14_H_8_O_9_). The characteristic fragment ions at 615.0638 and 309.0245 were generated through losing a C_13_H_9_O_9_ moiety, which implies the existence of the residue region composed of D- and E-rings. Compound **33** was tentatively attributed to phyllanthusiin C. The MS^2^ fragmentation pathway (Figure 4C) of phyllanthusiin C was proposed for the first time.

#### 2.1.5. Other Phenolics

Ten phenolics with a small molecular mass (<350 Da) and less than 15 carbons were tentatively identified in *P. capitatum.* The common fragment characteristic of phenolics is a successive or simultaneous loss of H_2_O and CO groups in their MS^2^ spectra. Compound **29** at t_R_ 5.58 min gave a deprotonated ion at *m/z* 247.0246 (C_12_H_7_O_6_^−^). The MS^2^ spectrum of compound **29** showed the fragment ions at *m/z* 219.0293 and 203.0343 resulting from the neutral loss of CO and CO_2_ unit from quasi-molecular ion, respectively. Furthermore, the ion at *m/z* 219.0293 went on losing a CO and a CO_2_ unit in succession to form the ions at 191.0342 and 147.0440. Since brevifolin has previously been found in *P. capitatum* in the reported literature [20], compound **29** was tentatively identified as brevifolin. Compound **46** gave a quasi-molecular ion at *m/z* 275.0197 ([M − H]^−^, C_13_H_7_O_7_^−^) and the fragment ions at *m/z* 257.0089 ([M − H − H_2_O]^−^), 247.0243 ([M − H − CO]^−^), 231.0291 ([M − H − CO_2_]^−^), 229.0138 ([M − H − H_2_O − CO]^−^) and 203.0343 ([M − H − CO − CO_2_]^−^). A lactone moiety or a carboxyl group was implied to be present in compound **46**. In addition, the ion at *m/z* 191.0341 was generated through an RDA fragmentation reaction from the ion at *m/z* 231.0291. Compound **46** was tentatively identified as urolithin M5. Urolithin M5 was an intestinal bacterial metabolite of ellagitannin davidiin from *P. capitatum* [26] and was also found in natural higher plants from diverse families [27,28]. The compound might be biosynthesized through the polyketide pathway [29,30] by endophytic fungi residing in raw *P. capitatum*. Compound **8** (t_R_ = 2.09 min) gave an [M − H]^−^ ion at *m/z* 243.0507 (C_10_H_11_O_7_^−^) and exhibited the characteristic ions of gallic acid moiety at *m/z* 169.0134, 125.0233 and 107.0125. The glycerol residue at *m/z* 91.0388 was found in the spectrum of compound **8**. Based on the reported literature [31], compound **8** was tentatively identified as galloyl-glycerol.

#### 2.1.6. Amino Acids and Amides

**Figure 5:**
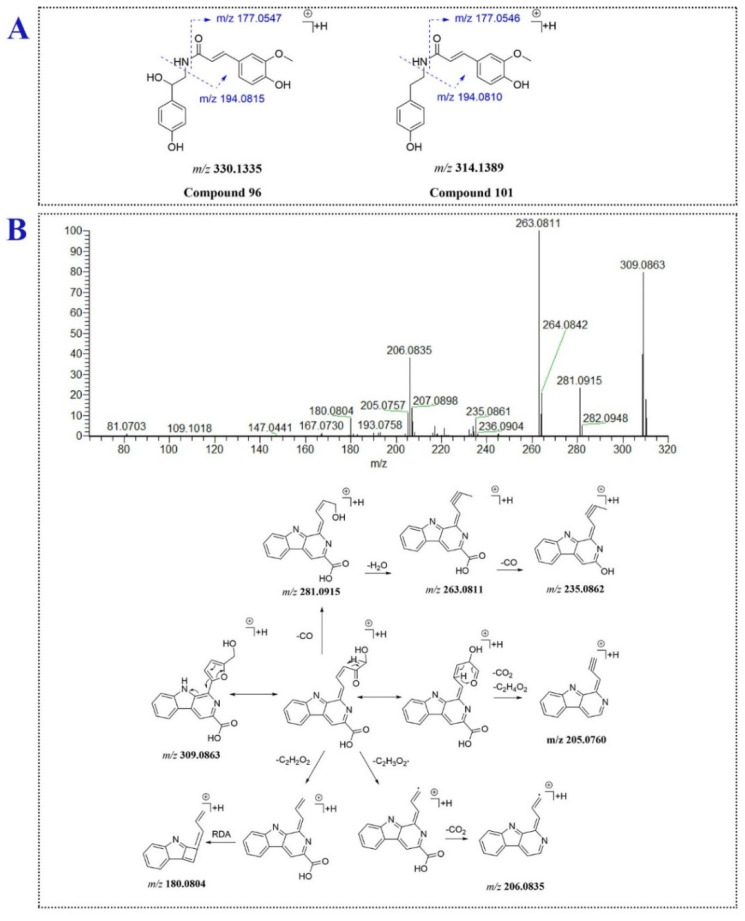
The fragmentation patterns of compounds **96**, **101** (**A**) and **106** (**B**).

Three Amino acids and 3 amides were identified or characterized in *P. capitatum.* Compound **6** (t_R_ = 2.03 min, [M + H]^+^, *m/z* 166.0864) was tentatively identified as the reported phenylalanine [12]. Compound **12** (t_R_ = 2.37 min) gave an [M + H]^+^ ion at *m/z* 328.1386 (C_15_H_23_O_7_N^+^) and lost a fructose moiety to form the characteristic ion of phenylalanine (*m/z* 166.0859 and 120.0808). Comparing the mass information of compound **12** to those in the reported literature [32], compound **12** was tentatively identified as fructose-phenylalanine. Compound **96** eluted at 15.82 min showed a quasi-molecular ion at *m/z* 330.1335 (C_18_H_20_NO_5_^+^) and gave the base-peak ion at *m/z* 177.0547 assigned to ferulic acid through neutral loss of an octopamine residue. The ions at *m/z* 145.0284 and 117.0338 were formed through successively losing CH_4_O and CO from the ion at *m/z* 177.0547. Moreover, the ion at *m/z* 194.0815 was generated through carbon-nitrogen bond cleavage (Figure 5A). Compound **96** was tentatively assigned as *N*-feruloyloctopamine. For compound **101** (t_R_ = 17.58 min, [M + H]^+^, C_18_H_20_NO_4_^+^), a tyramine moiety replaced the octopamine moiety compared to **96**. The MS^2^ data of compound **101** was similar to that of compound **96**. Compound **101** was tentatively assigned as *N*-feruloyltyramine (Figure 5A).

#### 2.1.7. Others

Compound **106** eluted at 17.80 min produced an [M + H]^+^ ion at *m/z* 309.0863 (C_17_H_13_O_4_N_2_^+^), suggesting compound **106** might be an alkaloid. In the MS/MS analysis, the fragment ions at *m/z* 281.0919, 263.0811 and 235.0862 were generated via ring fission and lost CO, H_2_O and CO units in succession. Furthermore, the [M + H]^+^ ion of compound **106** lost a C_2_H_2_O_2_ moiety of hydroxymethylfuran ring and underwent RDA fragment reaction to yield the fragment ion at *m/z* 180.0804. The product ion at *m/z* 206.0835 was formed though losing C_2_H_3_O_2_· and CO_2_ from the [M + H]^+^ ion. Compound **106** was tentatively assigned as flazin. The proposed mass fragment pathway of flazin was shown for the first time in the study (Figure 5B). Compound **41** (t_R_ = 7.67 min, [M − H]^−^, *m/z* 387.1660) generated the aglycone ion at *m/z* 207.1021 (C_12_H_15_O_3_^−^) through losing hexose sugar, and further lost one molecule of CO_2_ to form the ion at *m/z* 163.1118. Compound **41** was tentatively identified as the reported 12-hydroxyjasmonic acid glucoside [10].

### 2.2. UHPLC-Q-Orbitrap HRMS Analysis of the Prototype Compounds in Hyperuricemia Rat Plasma

The TIC chromatograms and mass data of rat plasma from hyperuricemia and drug-treated groups were compared to analyze *P. capitatum-*related exogenous components. The peaks that appeared at the same positions in the TIC chromatograms of both the dosed rat plasma and the herb extract but not in the chromatogram of the model rat plasma were regarded as prototype constituents. As a result, 16 prototype components of *P. capitatum* were found in hyperuricemia rat plasma. The detailed mass information is shown in Table S1. Among these, ellagic acid, 5,7-dihydroxychromone, quercetin-3-*O*-glucuronide, quercitrin, 3,3′-di-*O*-methylellagic acid, flazin, salidroside, 3,4,5-trimethoxyphenol-1-*O-β*-d-glucopyranoside, fructose-phenylalanine, nudiposide, quercetin-3*-O-β*-d-galactoside, quercetin-3-*O-β*-d-glucopyranoside, kaempferol-4-*O*′-rutinoside, *N*-feruloyltyramine and afzelin were found in rat plasma after oral administration of *P. capitatum* extracts for the first time.

### 2.3. UHPLC-Q-Orbitrap HRMS Analysis of P. capitatum Metabolites in Hyperuricemia Rat Plasma

The procedures for identification of metabolites included speculating probable metabolites according to the biotransformation rules of original compounds, extracting the [M − H]^−^ or [M + H]^+^ ions of probable metabolites from dosed plasma in full-scan mass mode and analyzing the MS^2^ information of the detected peak. The detected metabolic mechanism of *P. capitatum* in hyperuricemia rats involved ring fission, hydrolysis, decarboxylation, dehydroxylation, methylation, glucuronidation and sulfation. In this study, a total of 52 metabolites of *P. capitatum* in rat plasma were tentatively identified. Among them, 50 metabolites were revealed for the first time. The details of the characterized metabolites are listed in Table 1.

#### 2.3.1. Characterization of Phenolic-Related Metabolites

A total of 22 constituents were identified as phenolic-related metabolites, including 14 gallic acid-related (**M1–M3**, **M5–M6**, **M7–M12**, **M14**, **M20** and **M24**), 3 syringic acid/ethyl gallate-related (**M18**, **M19** and **M26**), 2 dimethylellagic acid-related (**M43** and **M47**), a vanillic acid-related (**M17**) and a protocatechuic acid-related (**M4**) metabolites.

**M18** (t_R_ = 5.67 min) and **M19** (t_R_ = 5.94 min) exhibited the same quasi-molecular ion at *m/z* 373.0775 ([M − H]^−^, C_15_H_17_O_11_^−^) and the same fragment ion at *m/z* 197.0448 by the cleavage of a dehydrated glucuronic acid moiety, suggesting the two metabolites were the glucuronidation products. Furthermore, **M18** showed the characteristic fragment ions at *m/z* 197.0448, 169.0144 and 125.0233 of ethyl gallate, while **M19** showed the fragment ions at *m/z* 197.0447, 182.0208, 166.9976 assigned to syringic acid. Therefore, **M18** and **M19** were identified as ethyl gallate glucuronide and syringic acid glucuronide, respectively. **M20** (t_R_ = 6.20 min) and **M26** (t_R_ = 7.02 min) exhibited the quasi-molecular ions [M-H]^−^ at *m/z* 277.0022 which were 79.9573 Da (SO_3_) more than that of 3,4-*O*-dimethylgallic acid or syringic acid and yielded the characteristic product ions of the two isomers at *m/z* 197.0449, 182.0213 and 166.9977. **M20** and **M26** were preliminarily assigned as 3,4-*O*-dimethylgallic acid sulfate or syringic acid-4-*O*-sulfate. To elucidate the exact conjugation site of **M20** and **M26**, their ClogP values were calculated through ChemBioDraw Ultra 20.0 software. Generally, compounds with larger ClogP values tend to form longer retention times in reverse-phase chromatography. The ClogP value of 3,4-*O*-dimethylgallic acid sulfate (−0.53863) is smaller than that of syringic acid-4-*O*-sulfate (−0.18863). Thus, **M20** and **M26** were speculated as 3,4-*O*-dimethylgallic acid sulfate and syringic acid-4-*O*-sulfate. **M2** and **M19** presented [M − H]^−^ ions at *m/z* 359.0620 (C_14_H_15_O_11_^−^) and lost a dehydrated glucuronic acid residue, a methyl group and a CO_2_ unit to form the ions at *m/z* 183.0292, 168.0068 and 124.0154, implying **M2** and **M19** were the methylated and glucuronidated products of gallic acid. Considering both 4-*O*-methylgallic acid and the 3-*O*-methylgallic acid were the main methylated metabolites of gallic acid [33] and 3-OH position was easier to be glucuronidated due to the smaller steric hindrance, **M2** with higher peak intensity was speculated as 4-*O*-methylgallic acid-3-*O*-glucuronide. **M19** was tentatively identified as 3-*O*-methylgallic acid-4-*O*-glucuronide. Similarly, **M7** lost an SO_3_ group to yield the aglycone ion assigned to methylated gallic acid. **M7** was tentatively identified as methylgallic acid sulfate.

**M43** (t_R_ = 13.95 min, *m/z* 505.0623, [M − H]^−^) and **M47** (t_R_ = 16.48 min, *m/z* 408.9866, [M − H]^−^) showed the same aglycone fragment ion at *m/z* 329.0303 through the loss of a dehydrated glucuronic acid and an SO_3_ unit, respectively. The ion at *m/z* 329.0303 discarded two methyl group in succession to yield the ions at *m/z* 314.0048 and 298.9833. The fragmentation behaviors of the aglycone ion at *m/z* 329.0303 was consistent with that of 3,3′-di-*O*-methylellagic acid. **M43** and **M47** was considered as 3,3′-di-*O*-methylellagic acid glucuronide and 3,3′-di-*O*-methylellagic acid sulfates.

#### 2.3.2. Characterization of Flavonoid-Related Metabolites

Six flavonols-related, 2 flavanone-related and 7 flavanols-related metabolites of *P. capitatum* were detected in dosed rat plasma. Sulfation, glucuronidation and methylation were the main metabolic pathways of the flavonoid. Moreover, ring fission and dehydroxylation were observed in the metabolic fate of flavanols (catechin or epicatechin).

**M44** and **M49** exhibited [M − H]^−^ ions at *m/z* 461.0726 (C_21_H_17_O_12_^−^) and 364.9972 (C_15_H_9_O_9_S^−^), respectively. The same fragment ions at *m/z* 285.0402 of **M44** and **M49** were corresponding to the loss of a dehydrated glucuronic acid and an SO_3_ group from their [M − H]^−^ ions, respectively. The ion at *m/z* 285.0402 furtherly lost a CH_2_O moiety to form the ion at *m/z* 255.0293. The fragment pattern of the aglycone ion was consistent with that of kaempferol in *P. capitatum* extract. According to the reported literature [34], **M44** and **M49** were identified as kaempferol glucuronide and kaempferol sulfate, respectively. **M41** (t_R_ = 13.07 min) produced a [M − H]^−^ ion at *m/z* 571.0396 (C_22_H_19_O_16_S^−^) and dissociated into the fragment ions at *m/z* 491.0832 (C_22_H_19_O_13_^−^) and 315.0511 (C_16_H_11_O_7_^−^) ascribed to the continuous losses of an SO_3_ unit and a glucuronic acid residue. The ion at *m/z* 315.0511 lost a methyl group to yield the product ion at *m/z* 300.0275 and furtherly was subjected to an RDA fragmentation reaction to form the ion at *m/z* 148.0155. The cracking path of the ion at *m/z* 315.0511 was similar to 3-*O*-methylquercetin [35]. **M41** was speculated as glucuronidation and sulfation of 3-*O*-methylquercetin.

**Figure 6:**
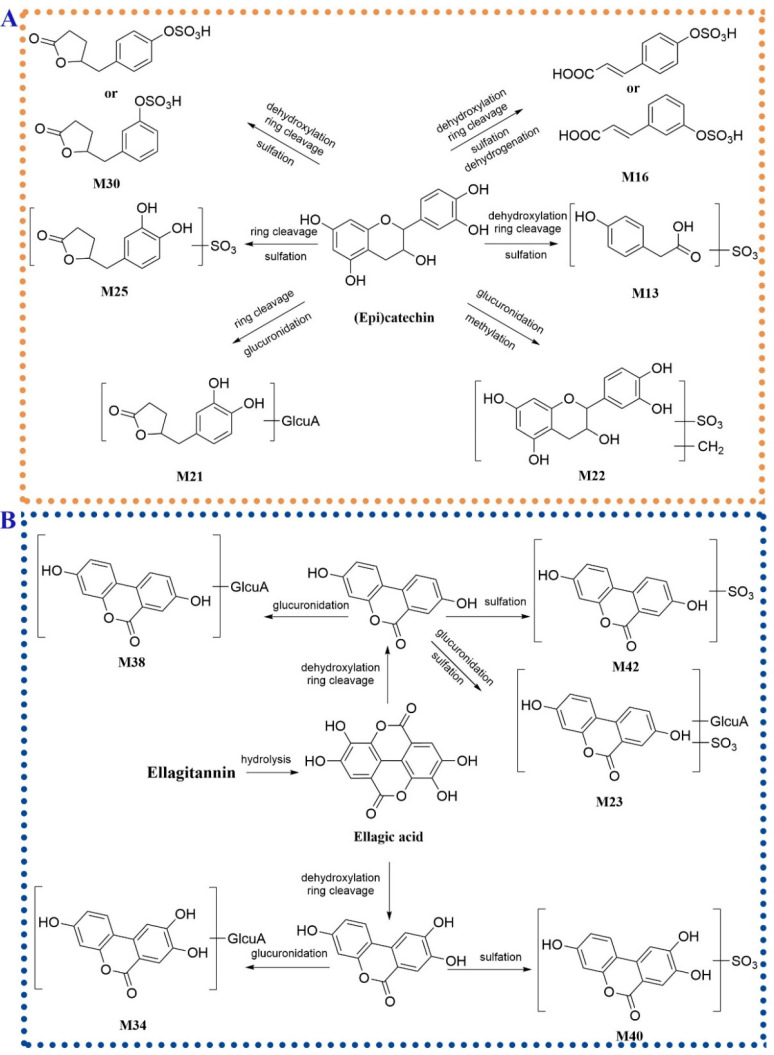
The possible metabolic pathways of (epi)catechin (**A**) and ellagitannins (**B**) in hyperuricemia rats administered orally with *P. capitatum* extract.

Ring cleavage was a common metabolic pathway of (epi)catechin in vivo [36,37]. These metabolites generated through ring cleavage were further bio-transformed through sulfation or glucuronidation. **M13** (t_R_ = 4.32 min) showed an [M − H]^−^ ion at *m/z* 230.9964 (C_8_H_7_O_6_S^−^) and an aglycone ion at 151.0393 through loss of an SO_3_ unit. The [M − H]^−^ ion produced the fragment ions at *m/z* 187.0063 (C_7_H_7_O_4_S^−^) and 107.0490 (C_7_H_7_O^−^) by discarding a CO_2_ group and an SO_3_ group in succession, which was in high accordance with mass fragment pattern of 3-hydroxyphenylacetic acid sulfate [36]. **M13** was considered the metabolite of (epi)catechin through dehydroxylation, ring cleavage and sulfation (3-hydroxyphenylacetic acid sulfate). **M25** (t_R_ = 6.88 min) showed an [M − H]^−^ ion at *m/z* 287.0233 (C_11_H_11_O_7_S^−^) and an [aglycone − H]^−^ ion at *m/z* 207.0504 generated by the loss of an SO_3_ moiety. The ion at *m/z* 163.0764 was yielded from the [aglycone − H]^−^ ion through losing a CO_2_ unit. According to the reported metabolic pathway of (epi)catechin [38], **M25** was tentatively characterized as 5-(3′,4′-dihydroxyphenyl)-γ-valerolactone sulfate. Compared to **M25**, **M30** exhibited the absence of an oxygen atom (16 Da) and a similar fragment pattern to **M25**. **M30** was tentatively assigned as 5-(3′-hydroxyphenyl)-γ-valerolactone sulfate or 5-(4′-hydroxyphenyl)-γ-valerolactone sulfate. Similarly, 5-(3′,4′-hydroxyphenyl)-γ-valerolactone glucuronide (**M21**, C_17_H_19_O_10_^−^) was tentatively identified in spite of no fragment ions obtained in the MS^2^ spectrum [36]. **M16** (t_R_ = 5.50 min) produced a [M − H]^−^ ion at *m/z* 242.9966 (C_9_H_7_O_6_S^−^). In the MS^2^ spectrum, the fragment ions at *m/z* 163.0392 ([M − H − SO_3_]^−^), 135.0443 ([M − H − SO_3_ − CO]^−^) and 119.0491 ([M − H − SO_3_ − CO_2_]^−^) were consistent with the fragment pathway of *m*-coumaric acid or *p*-coumaric acid [39]. **M16** was tentatively assigned as *m*-coumaric acid sulfate or *p*-coumaric acid sulfate. Based on the above data, the possible metabolic pathways of (epi)catechin in hyperuricemia rats administered orally with *P. capitatum* extract are shown in Figure 6A.

#### 2.3.3. Characterization of Phenylpropanoid-Related Metabolites

A total of 8 phenylpropanoid-related metabolites were detected in rat plasma, mainly from the products of isolariciresinol and 5,7-dihydroxychromone. **M31** (t_R_ = 8.39 min), **M32** (t_R_ = 8.55 min), **M35** (t_R_ = 9.65 min) and **M36** (t_R_ = 9.86 min) showed the same quasi-molecular ion [M − H]^−^ at *m/z* 535.1823 (C_26_H_31_O_12_^−^) and gave the fragment ions at *m/z* 359.1497 ([M − H − glucuronyl unit]^−^), 344.1266 ([M − H − glucuronyl unit − CH_3_·]^−^) and 329.1036 ([M − H − glucuronyl unit − 2 × CH_3_·]^−^) in their MS^2^ spectra. The ion at *m/z* 241.0507 was formed from the ion at *m/z* 329.1036 through an RDA fragmentation pathway. **M31**, **M32**, **M35** and **M36** were preliminarily assigned as glucuronidation of isolariciresinol. The conjugation sites of the four metabolites were speculated by ClogP values. **M31** (ClogP = −1.3851) and **M32** (ClogP = −1.3851) were tentatively identified as isolariciresinol-4 (or 4′)-*O*-glucuronide. **M35** (ClogP = −0.7404) and **M36** (ClogP = −0.7404) were tentatively identified as isolariciresinol-9 (or 9′)-*O*-glucuronide.

#### 2.3.4. Characterization of Tannis-Related Metabolites

According to the reported literature, although ellagitannins were not absorbed in vivo, ellagitannins located at the distal segment of the gastrointestinal tract could be bio-transformed by the intestinal bacteria into dibenzo-*α*-pyrones derivatives [40]. Compared to the base peak chromatography (BPC) of the plasma from the hyperuricemia group, a peak with high intensity at 10.13 min (**M38**) was detected in the BPC of dosed rat plasma. **M38** exhibited an [M − H]^−^ ion at *m/z* 403.0669 (C_19_H_15_O_10_^−^) and lost a dehydrated glucuronic acid residue to form the ions at *m/z* 227.0345 ([M − H − glucuronyl unit]^−^) and 175.0239 (glucuronyl unit), suggesting the **M38** was a glucuronic conjugate. The aglycone ion at *m/z* 227.0345 furtherly lost a CO group and a CO_2_ unit to generate the fragment ions at *m/z* 199.0388 and 183.0446, respectively. The ion at *m/z* 155.0491 was formed through the combined loss of CO and CO_2_ from the aglycone ion. The fragment pattern of the aglycone ion was consistent with that of urolithin A [41]. **M38** was tentatively identified as urolithin A glucuronide. **M42** (*m/z* 306.9917, [M − H]^−^, C_13_H_7_O_7_S^−^) lost an SO_3_ group to yield the aglycone ion at *m/z* 227.0345 assigned to urolithin A. **M42** was identified as urolithin A sulfate. Similarly, **M23** (t_R_ = 6.74 min) lost an SO_3_ group and a dehydrated glucuronic acid moiety to form the aglycone fragment of urolithin A. **M23** was speculated as glucuronidation and sulfation product of urolithin A. Compared to the quasi-molecular ions and the corresponding fragment ions of **M38** and **M42**, **M34** and **M40** showed an additional oxygen atom (15.9951 Da). **M34** and **M40** were identified as urolithin C glucuronide and urolithin C sulfate, respectively. The possible metabolic pathways of ellagitannins in hyperuricemia rats orally administered with *P. capitatum* were shown in Figure 6B.

#### 2.3.5. Characterization of Alkaloid-Related Metabolites

**M52** (t_R_ = 20.68 min) showed an [M + H]^+^ ion at *m/z* 323.1024 (C_18_H_15_O_4_N_2_^+^) that was more 14.0161 Da (CH_2_ moiety) than that of flazin. The MS^2^ spectrum of **M52** in positive ion mode showed the fragment ions at *m/z* 263.0814, 206.0837 and 180.0806 assigned to flazin as discussed above. Furthermore, **M52** detected at *m/z* 321.0880 ([M − H]^−^) in negative ion mode yielded the fragment ions at 291.0775 and 277.0612 by losing CH_2_O and C_2_H_4_O, suggesting the existence of a methoxy group in **M52**. The ion at *m/z* 277.0612 furtherly lost a CO_2_ group and a molecule of H_2_O to form the ion at *m/z* 233.0719 and 259.0511. **M52** was tentatively identified as flazin methyl ether.

## 3. Materials and Methods

### 3.1. Material and Reagents

The herb of *P. capitatum* was collected from Qianxi county, Guizhou province, China and was identified by Qingde Long (Guizhou Medical University) as the whole plant of *Polygonum capitatum* Buch.-Ham. ex D. Don. The voucher specimen of *P. capitatum* (No.: PC20201103) was deposited in the Herbarium of Guizhou Medical University.

The reference standards of gallic acid, protocatechuic acid, (+)-catechin, rutin, quercitrin, quercetin and emodin were purchased from Chengdu Chroma-Biotechnology Co., Ltd. (Chengdu, China). The pure materials of myricitrin, quercetin-3-*O*-(2″-*O*-galloyl)-*β*-d-glucopyranoside, quercetin-3-*O*-*β*-d-glucopyranoside, *cis*-*N-*caffeoyltyramine and 3″-*O*-galloylquercitrin were obtained in our laboratory. The purities of the reference standards were determined to be more than 98% by HPLC (-DAD. Hypoxanthine and potassium oxonate were purchased from the Beijing Solarbio Science and Technology Co., Ltd. (Beijing, China). Urate assay kits were purchased from Nanjing Jiancheng Bioengineering Institute (Nanjing, China).

HPLC-grade methanol and acetonitrile were acquired from Honeywell Burdick & Jackson Company (Morristown, NJ, USA). Formic acid (MS grade) was obtained from Fisher Scientific (Madrid, Spain). Deionized water for HPLC analysis was prepared using a Milli-Q water purification system (Millipore, Milford, MA, USA). All other reagents were of analytical grade.

### 3.2. Preparation of Mixed Standard Solutions

The stock solutions of standards were prepared by weighting appropriate amounts of 13 reference substances individually and dissolving them in methanol at a concentration of 1.0 mg/mL. The final mixed standard solution (200 ng/mL) was obtained by mixing the appropriate volumes of the stock solutions and diluting with 60% methanol before qualitative analysis.

### 3.3. Preparation of P. capitatum Samples

The dried raw herb of *P. capitatum* (1453 g) was weighed and crushed into power. The obtained powder was immersed in a ten-fold volume of distilled water for 30 min and decocted three times by boiling for 1 h. The decoctions were filtered to remove the herbal residue. The supernatants were merged together and concentrated to yield the extract residue (291.1 g, the extraction rate 20.03%).

10 mg of the obtained extract residue was dissolved in 1 mL of 60% (*v*/*v*) methanol and ultrasonicated for 30 min at 100 kHz. After centrifuging at 12,000 rpm for 10 min, 10 µL of the supernatant was used for UHPLC-Q-Orbitrap HRMS analysis.

### 3.4. Animal Treatment and Drug Administration

A total of 18 male Sprague-Dawley (SD) rats (weighing 200 ± 20 g) were obtained from Changsha Tianqin Biotechnology Company (Hunan, China). The rats were housed under a standard 12-h light-dark cycle at 25 ± 2 °C and 60 ± 5% humidity with free access to water and a normal diet for 7 days. All of the experiments were approved by the Animal Care Welfare Committee of Guizhou Medical University (approval number 2100138) and performed according to the Guide for the Care and Use of Laboratory Animals (National Institutes of Health).

Rats were randomly divided into three groups with six animals per group (control, hyperuricemia and drug-treated groups). Hyperuricemia was induced in the rats according to the described method previously [42]. Briefly, except for the control group, intragastric hypoxanthine (500 mg/kg/day) and intraperitoneal injection of potassium oxonate (100 mg/kg/day) were given to the rats for 7 days. The animals in the control group received physiological saline in a similar fashion. On the 4th day of hyperuricemia induction, the *P. capitatum* extract (5 g/kg/day) was administered orally to the rats in the drug-treated group at 1 h after dosing of the modeling agents for 3 days. The serum urate levels in the control and hyperuricemia rat groups were detected by urate assay kits during the experimental period. The serum urate levels in the hyperuricemia rat group significantly increased compared to those of the control rats (*p* < 0.05), indicating the successful establishment of the hyperuricemia model.

### 3.5. Collection and Preparation of Plasma Sample

Blood samples were collected at 0.083, 0.25, 0.5, 1, 2, 4, 6, 8, 12 and 24 h after the last administration from the retro-orbital plexus into heparinized tubes. Plasma was obtained through centrifugation at 3000 rpm for 10 min. All plasma samples at different time points from each group of rats were combined to produce the pooled sample for eliminating the individual variability. A 500 µL volume of the pooled plasma sample was added with 1.5 mL of acetonitrile and vortexed for 1.0 min to precipitate protein. The sample was centrifuged at 12,000 rpm and 4 °C for 10 min. The supernatant was evaporated to dryness under a gentle flow of nitrogen at room temperature. The residue was redissolved with 200 µL of 60% methanol in water and centrifuged at 12,000 rpm for 10 min. The supernatant was injected into the UHPLC-Q-Orbitrap HRMS system for analysis.

### 3.6. UHPLC-Q-Orbitrap HRMS Conditions

A Dionex Ultimate 3000 UHPLC system (Thermo Fisher Scientific, Waltham, MA, USA) consisted of a quaternary solvent delivery system, a column compartment and a refrigerated auto-sampler. The sample separation was performed on an ACQUITY UPLC^®^ BEH C18 column (2.1 mm × 100 mm, 1.7 µm) eluted with acetonitrile (A) and 0.1% aqueous formic acid (B). The flow rate was set at 0.3 mL/min with an initial mobile phase of 5% (A). The chromatographic elution program was set: 5–5% A at 0–1.0 min, 5–10% A at 1.0–4.0 min, 10–12% A at 4.0–9.0 min, 12–20% A at 9.0–14.0 min, 20–45% A at 14.0–19.0 min, 45–70% A at 19.0–20.0 min, 70–100% A at 20.0–22.0 min, 100–5% A at 22.0–22.1 min, 5–5% A at 22.1–25.0 min. The injection volume was 10 µL.

A Q-Exactive™ Orbitrap mass spectrometer (Thermo Fisher Scientific, Waltham, MA) equipped with a heated electrospray ionization source (HESI) was used for qualitative analysis. The analysis was carried out both in positive and negative ion modes. The collision and nebulizing gases were ultra-high purity helium (He) and high purity nitrogen (N_2_). The parameters were set as follows: ion spray voltage: +3.0 kV and −2.5 kV, capillary temperature: 320 °C, S-lens RF level: 60%. The flow rates of sheath gas and auxiliary gas were set to 35 and 10 arbitrary units, respectively. A full MS/dd-MS^2^ acquisition program was executed with resolutions of 70,000 and 17,500 FWHM. For the full MS experiments, the scan range was from 80 to 1200 *m/z*, the automatic gain control (AGC) target was defined as 1e^6^ and the maximum injection time (IT) was set as auto. For the dd-MS^2^ experiments, AGC target: 2e^5^, maximum IT: auto, loop count: 1, the isolation window was 3.0 *m/z*. The stepped normalized collision energies (NCE) were 20, 40, and 60 eV.

### 3.7. Data Analysis

**Figure 7:**
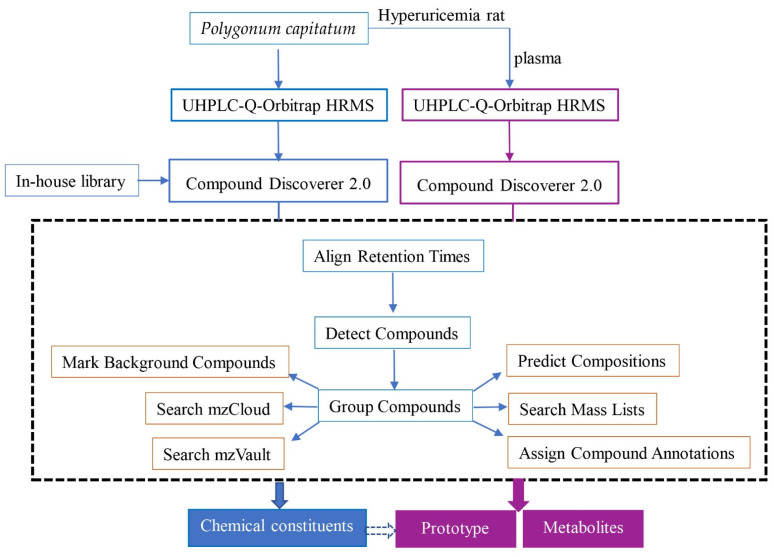
The data processing workflow for identification of chemical ingredients from *P. capitatum* and its absorbed constituents in hyperuricemia rat plasma.

The information of chemical constituents from *P. capitatum*, including CAS number, molecular formula and molecular weight, were obtained by retrieving SciFinder Scholar and Dictionary of Natural Product databases. An in-house library containing potential compounds from *P. capitatum* extract was established. Data analysis was performed through Xcalibur 3.0 software (Thermo Fisher Scientific, Waltham, MA, USA) and Compound Discoverer 2.0 software coupled to mzCloud© and ChemSpider© databases. The data processing workflow for the identification of chemical ingredients from *P. capitatum* and its metabolites was shown in Figure 7.

## 4. Conclusions

In this study, a sensitive and accurate UHPLC-Q-Orbitrap HRMS method was utilized to systematically analyze the chemical constituents of *P. capitatum* and its absorbed components in hyperuricemia rats. A total of 114 compounds including phenolic acids, flavonoids, phenylpropanoids, tannins, phenolics, amino acids, amides and others were identified or characterized. At the same time, 68 *P. capitatum*-related xenobiotics were found in the hyperuricemia rats’ plasma. These exogenous components in hyperuricemia rats might be the potential active constituents of *P. capitatum* for anti-hyperuricemia and anti-gouty arthritis. The detected metabolic pathway of *P. capitatum* in hyperuricemia rats included ring fission, hydrolysis, decarboxylation, dehydroxylation, methylation, glucuronidation and sulfation. This study not only supplied a basis for the further investigation of the active components and pharmacokinetics of *P. capitatum*, but also provided insight into the anti-hyperuricemia mechanism and quality control of *P. capitatum*.

## Figures and Tables

**Table 1:** The metabolites of *P. capitatum* in hyperuricemia rat plasma.

Peak No.	Compounds	Molecular Formula	t_R_	Ion Mode	Precursor Ion	Error (ppm)	Product Ions
M1	Gallic acid glucuronide	C_13_H_14_O_11_	1.93	negative mode	345.0464	3.25	169.0132
M2	4-*O*-methylgallic acid-3-*O*- glucuronide	C_14_H_16_O_11_	1.93	negative mode	359.0620	2.96	183.0292, 168.0068, 124.0154
M3	Gallic acid sulfate	C_7_H_6_O_8_S	2.13	negative mode	248.9707	3.15	169.0132
M4	Protocatechuic acid sulfate	C_7_H_6_O_7_S	2.23	negative mode	232.9757	2.62	153.0183, 109.0295
M5	2-*O*-Methylpyrogallol sulfate	C_7_H_8_O_6_S	2.35	negative mode	218.9963	2.31	139.0389, 124.0154
M6	1-*O*-Methylpyrogallol-3-*O*-sulfate	C_7_H_8_O_6_S	2.72	negative mode	218.9964	2.72	139.0390, 124.0154
M7	Methylgallic acid sulfate	C_8_H_8_O_8_S	2.75	negative mode	262.9866	3.63	183.0291, 168.0055
M8	Pyrogallol-1-*O*-sulfate or Pyrogallol-2-*O*-sulfate	C_6_H_6_O_6_S	2.85	negative mode	204.9806	2.46	125.0233
M9	2-*O*-methylpyrogallol-1-*O*- glucuronide	C_13_H_16_O_9_	3.02	negative mode	315.0725	4.51	139.0401, 124.0166, 113.0245
M10	4-*O*-methylgallic acid or 3-*O*-methylgallic acid	C_8_H_8_O_5_	4.04	negative mode	183.0291	1.64	168.0055, 124.0155
M11	Phenol sulfate	C_6_H_6_O_4_S	4.08	negative mode	172.9905	1.24	93.0333
M12	Pyrogallol-1-*O*-glucuronide or Pyrogallol-2-*O*-glucuronide	C_12_H_14_O_9_	4.28	negative mode	301.0564	3.39	125.0232
M13	Dehydroxylation and ring cleavage and sulfation of catechin (3-hydroxyphenylacetic acid sulfate)	C_8_H_8_O_6_S	4.32	negative mode	230.9964	2.79	187.0063, 151.0393, 107.0490, 79.9560
M14	1-*O*-Methylpyrogallol-2-*O*-sulfate	C_7_H_8_O_6_S	4.74	negative mode	218.9968	4.82	139.0402, 124.0166
M15	Glucuronidation of 5,7- Dihydroxychromone	C_15_H_14_O_10_	5.35	negative mode	353.0510	2.00	177.0183
M16	*m*-Coumaric acid sulfate or *p*-coumaric acid sulfate	C_9_H_8_O_6_S	5.50	negative mode	242.9966	3.27	163.0392, 119.0493
M17	Sulfation of vanillic acid	C_8_H_8_O_7_S	5.63	negative mode	246.9914	2.92	167.0340, 152.0107,
M18	Ethyl gallate glucuronide	C_15_H_18_O_11_	5.67	negative mode	373.0775	2.61	197.0448, 169.0144, 125.0233
M19	Syringic acid glucuronide	C_15_H_18_O_11_	5.94	negative mode	373.0775	2.61	197.0447, 182.0208, 166.9976
M20	3,4-*O*-dimethylgallic acid sulfate	C_9_H_10_O_8_S	6.20	negative mode	277.0022	3.49	197.0449, 182.0213, 166.9977
M21	5-(3′,4′-Hydroxyphenyl)-*γ*-valerolactone glucuronide	C_17_H_20_O_10_	6.46	negative mode	383.0979	1.64	no fragment
M22	Methylcatechin glucuronide	C_22_H_24_O_12_	6.64	negative mode	479.1188	0.91	465.1025, 303.0863
M23	Urolithin A glucuronide-sulfate diconjugate	C_19_H_16_O_13_S	6.74	negative mode	483.0233	1.10	403.0664, 227.0346
M24	3-*O*- methylgallic acid-4-*O*-glucuronide	C_14_H_16_O_11_	6.81	negative mode	359.0617	2.29	183.0293, 168.0068, 124.0154
M25	5-(3′,4′-Dihydroxyphenyl)-γ-valerolactone sulfate	C_11_H_12_O_7_S	6.88	negative mode	287.0233	4.35	207.0504, 163.0764, 79.9574
M26	Syringic acid-4-*O*-sulfate	C_9_H_10_O_8_S	7.02	negative mode	277.0021	3.16	197.0454, 182.0213, 166.9976
M27	Quercitrin glucuronide	C_27_H_28_O_17_	7.20	negative mode	623.1252	1.55	447.0935
M28	Sulfation of 5,7- Dihydroxychromone	C_9_H_6_O_7_S	7.41	negative mode	256.9760	3.85	177.0186, 133.0284
M29	Trihydroxyflavanone glucuronide	C_22_H_20_O_12_	7.60	negative mode	463.0884	2.76	287.0561
M30	5-(3′-Hydroxyphenyl)-γ- valerolactone sulfate or 5-(4′-hydroxyphenyl)-γ- valerolactone sulfate	C_11_H_12_O_6_S	8.09	negative mode	271.0280	3.19	191.0706, 147.0820
M31	Isolariciresinol-4 (or 4′)-*O*-glucuronide	C_26_H_32_O_12_	8.39	negative mode	535.1819	1.64	359.1497, 344.1266, 329.1036, 241.0507
M32	Isolariciresinol-4 (or 4′)-*O*-glucuronide	C_26_H_32_O_12_	8.55	negative mode	535.1822	2.21	359.1498, 344.1259
M33	Glucuronidation of 3,5′-dimethoxy-isolariciresinol	C_28_H_36_O_14_	8.72	negative mode	595.2025	-0.07	419.1709, 404.1469
M34	Urolithin C glucuronide	C_19_H_16_O_11_	9.47	negative mode	419.0620	2.61	243.0300
M35	Isolariciresinol-9 (or 9′)-*O*- glucuronide	C_26_H_32_O_12_	9.65	negative mode	535.1821	2.00	359.1498
M36	Isolariciresinol-9 (or 9′)-*O*- glucuronide	C_26_H_32_O_12_	9.86	negative mode	535.1823	2.44	359.1504, 344.1267, 329.1029, 241.0507
M37	Sulfation of Isolariciresinol	C_20_H_24_O_9_S	10.00	negative mode	439.1073	2.19	359.1497
M38	Urolithin A glucuronide	C_19_H_16_O_10_	10.13	negative mode	403.0669	2.20	227.0345, 199.0388, 183.0446, 175.0239, 155.0491
M39	Quercetin diglucuronide	C_27_H_26_O_19_	11.42	negative mode	653.0994	0.98	447.0671, 301.0345
M40	Urolithin C sulfate	C_13_H_8_O_8_S	12.80	negative mode	322.9865	2.87	243.0297
M41	3-*O*-Methylquercetin glucuronide sulfate	C_22_H_20_O_16_S	13.07	negative mode	571.0396	1.57	491.0832, 315.0511, 300.0275, 148.0155
M42	Urolithin A sulfate	C_13_H_8_O_7_S	13.60	negative mode	306.9917	1.03	227.0346, 199.0398, 183.0444
M43	3,3′-Di-*O*-methylellagic acid glucuronide	C_22_H_18_O_14_	13.95	negative mode	505.0623	2.10	329.0303, 314.0048, 298.9833, 270.9883
M44	Kaempferol glucuronide	C_21_H_18_O_12_	15.11	negative mode	461.0726	2.42	285.0402, 255.0293
M45	Methylation of Ellagic acid	C_15_H_8_O_8_	15.82	negative mode	315.0146	3.35	299.9910
M46	Naringenin glucuronide	C_21_H_20_O_11_	15.82	negative mode	447.0934	1.22	271.0612
M47	3,3′-Di-*O*-methylellagic acid sulfate	C_16_H_10_O_11_S	16.48	negative mode	408.9866	1.52	329.0304, 314.0062, 298.9833
M48	Sulfation and loss of 2 × oxygen of catechin	C_15_H_14_O_6_S	16.86	negative mode	321.0436	2.73	241.0866, 147.0440, 135.0440, 121.0283
M49	Kaempferol sulfate	C_15_H_10_O_9_S	17.51	negative mode	364.9972	2.77	285.0403, 255.0294
M50	Methylquercetin glucuronide	C_22_H_20_O_13_	18.03	negative mode	491.0829	1.78	315.0516, 300.0273
M51	Methylation of Procyanidin B1 or Procyanidin B2	C_31_H_28_O_12_	20.28	negative mode	591.1525	4.26	no fragment
M52	Flazin methyl ether	C_18_H_14_O_4_N_2_	20.68	positive mode	323.1024	−0.85	263.0814, 206.0837, 180.0806
				negative mode	321.0879	2.86	259.0511, 217.0766

## Supplementary Materials

The following supporting information can be downloaded at: https://www.mdpi.com/article/10.3390/molecules27113521/s1, Table S1: Chemical constituents identified and characterized in *P. capitatum* by UHPLC-Q-Orbitrap HRMS in negative and positive ion modes. Figure S1: The chemical structures of the constituents from *P. capitatum* analyzed by UHPLC-Q-Orbitrap HRMS.

## Sample Availability

Samples of the compounds are not available from the authors.
