# Supplementary material for: Systematically Exploring the Chemical Ingredients and Absorbed Constituents of Polygonum capitatum in Hyperuricemia Rat Plasma Using UHPLC-Q-Orbitrap HRMS
Source: Molecules. 2022 May 30;27(11):3521. doi: 10.3390/molecules27113521 (PMC9182448; doi:10.3390/molecules27113521)
Supplement: Supplementary file 1 [file molecules-27-03521-s001.zip › molecules-1710266-supplementary.pdf]

Systematically exploring the chemical ingredients and absorbed constituents of *Polygonum capitatum* in hyperuricemia rat plasma using UHPLC-Q-Orbitrap HRMS

Huanyu Guan [1,†] , Pengfei Li [2,†], Qian Wang [1], Fanli Zeng [1], Daoping Wang [1,3], Mei Zhou [1,3], Meng Zhou [1], Xun He [1], Shanggao Liao [1,*], Weidong Pan [1,3,*]

[1] State Key Laboratory of Functions and Applications of Medicinal Plants & School of Pharmacy, Guizhou Medical University, Guiyang 550025, China

[2] National Institute of Drug Clinical Trial, Guizhou Provincial People's Hospital, Guiyang 550002, China

[3] Key Laboratory of Chemistry for Natural Products of Guizhou Province and Chinese Academy of Sciences, Guiyang 550014, China

*Correspondence: sgliao@gmc.edu.cn (S.L.); weidongpan@gzcnp.cn (W.P.).

† These authors contributed equally to this work

Supplementary Materials

- **Table S1** Chemical constituents identified and characterized in *P. capitatum* by UHPLC-Q-Orbitrap HRMS in negative and positive ion modes
- **Figure S1** The chemical structures of the constituents from *P. capitatum* analyzed by UHPLC-Q-Orbitrap HRMS

Table S1. Chemical constituents identified and characterized in *P. capitatum* by UHPLC-Q-Orbitrap HRMS in negative and positive ion modes.

Peak No.	Compounds	Molecular formula	t_R	Ion mode	Precursor ion	Error (ppm)	Product ions
1	3-O-Galloyl-*α*-D-glucopyranoside or 3-*O*-galloyl-*β*-D-glucopyranoside	$C_{13}H_{16}O_{10}$	1.23	negative mode	331.0669	2.89	271.0459, 211.0241, 169.0133, 125.0232
2	3-O-Galloyl-*α*-D-glucopyranoside or 3-*O*-galloyl-*β*-D-glucopyranoside	$C_{13}H_{16}O_{10}$	1.41	negative mode	331.0669	2.89	271.0459, 211.0242, 169.0133, 125.0232
3	5-Hydroxymethylfurfural	$C_6H_6O_3$	1.45	negative mode	125.0232	-1.04	107.0125, 97.0281
				positive mode	127.0390	-0.08	109.0286, 99.0444, 85.0593
4*#	Gallic acid	$C_7H_6O_5$	1.60	negative mode	169.0133	1.00	125.0233, 107.0126, 97.0283
5	Tachioside or isotachioside or picraquassioside D	$C_{13}H_{18}O_8$	1.66	negative mode	301.0927	2.94	139.0388,121.0283
6	Phenylalanine	$C_9H_{11}O_2N$	2.03	negative mode	164.0707	0.70	147.0441
				positive mode	166.0864	0.63	120.0809
7	Tachioside or isotachioside or picraquassioside D	$C_{13}H_{18}O_8$	2.04	negative mode	301.0926	2.64	/
8	Galloyl-glycerol	$C_{10}H_{12}O_7$	2.09	negative mode	243.0507	3.17	169.0134, 125.0233, 107.0125
9	Vanillic acid	$C_8H_8O_4$	2.20	negative mode	167.0341	1.29	152.0103, 123.0440, 108.0206, 93.0333
10	Dihydroxybenzoic acid	$C_7H_6O_4$	2.26	negative mode	153.0183	0.49	125.0232, 109.0282
11	3,5-Dimethoxy-4-hydroxyphenol-1-*O*-*β*-D-glucopyranoside	$C_{14}H_{20}O_9$	2.28	negative mode	331.1033	2.84	/
12#	Fructose-phenylalanine	$C_{15}H_{22}O_7N$	2.37	negative mode	326.1245	3.23	164.0708, 147.0441
				positive mode	328.1386	-1.34	310.1277, 292.1172, 264.1224, 166.0859, 120.0808
13	Vanillic acid-*O*-hexoside	$C_{14}H_{18}O_9$	2.52	negative mode	329.0858	-2.70	167.0341, 152.0105, 123.0440, 108.0205
14*	Protocatechuic acid	$C_7H_6O_4$	2.58	negative mode	153.0183	0.49	109.0283
15	2,5-Dihydroxybenzoic acid	$C_7H_6O_4$	2.95	negative mode	153.0184	0.82	109.0283
16	Tachioside or isotachioside or picraquassioside D	$C_{13}H_{18}O_8$	2.97	negative mode	301.0928	3.34	139.0390, 121.0283
17	Syringic acid-*O*-hexoside	$C_{15}H_{20}O_{10}$	3.20	negative mode	359.0984	3.01	197.0448, 182.0211, 153.0547, 138.0311
				positive mode	361.1122	-1.98	/
18	L-tryptophan	$C_{11}H_{12}O_2N_2$	3.51	negative mode	203.082	2.29	159.0918, 142.0651, 116.0494
				positive mode	205.0970	-0.51	188.0703, 170.0597, 146.0598

19	Ethyl protocatechuate	$C_9H_{10}O_4$	3.64	negative mode	181.0498	1.68	153.0183, 109.0283, 108.0203,
20	3-O-(3,4,5-Trihydroxybenzoyl)-1-*O*,6-*O*-[2,2',3,3',4,4'-hexahydroxy(1,1'-biphenyl)-6,6'-diylbiscarbonyl]-beta-D-glucopyranose (CAS130233-85-1)	$C_{27}H_{22}O_{18}$	3.64	negative mode	633.0729	1.11	300.9990, 275.0194
21	Digalloyl-glucopyranose	$C_{20}H_{20}O_{14}$	4.04	negative mode	483.0778	1.84	313.0565, 271.0457, 211.0241, 160.0133, 125.0232
22	4-Hydroxybenzoic acid	$C_7H_6O_3$	4.05	negative mode	137.0234	0.51	119.0127, 108.02204, 93.0333
				positive mode	139.0388	-0.87	111.0442
23#	Salidroside	$C_{14}H_{20}O_7$	4.31	negative mode	299.1134	2.91	/
24#	3,4,5-trimethoxyphenol-1-*O*-*β*-D-glucopyranoside	$C_{15}H_{22}O_9$	4.33	negative mode	345.1189	2.58	179.0553
25	Procyanidin B1 or procyanidin B2	$C_{30}H_{26}O_{12}$	4.65	negative mode	577.1348	1.35	451.1034, 425.0877, 407.0769, 289.0717
				positive mode	579.1498	0.24	409.0909, 163.0387, 127.0390
26*	(+)-Catechin	$C_{15}H_{14}O_6$	5.01	negative mode	289.0717	3.76	271.0613, 245.0817, 151.0335, 125.0233, 109.0283
				positive mode	291.0858	-1,84	139.0389, 123.0441
27	6'-*O*-Galloylarbutin	$C_{19}H_{20}O_{11}$	5.18	negative mode	423.0933	2.68	313.0565, 169.0134, 125.0233
28	3-Methoxy-4-hydroxyphenol-1-*O*-*β*-D-(6'-*O*-galloyl)glucopyranoside or 2-methoxy-4-hydroxyphenol-1-*O*-*β*-D-(6'-*O*-galloyl)glucopyranoside or 3-methoxy-5-hydroxyphenol-1-*O*-*β*-D-(6'-*O*-galloyl)glucopyranoside	$C_{20}H_{22}O_{12}$	5.54	negative mode	453.1037	2.16	313.0560, 169.0133
29	Brevifolin	$C_{12}H_8O_6$	5.58	negative mode	247.0246	3.38	219.0293, 203.0343, 191.0342,147.0440
30	Brevifolin carboxylic acid	$C_{13}H_8O_8$	5.91	negative mode	291.0146	3.80	247.0245, 219.0293, 119.0341
				positive mode	293.0289	-0,90	275.0183, 249.0394
31	3-Methoxy-4-hydroxyphenol-1-*O*-*β*-D-(6'-O-galloyl)glucopyranoside or 2-methoxy-4-hydroxyphenol-1-O-*β*-D-(6'-*O*-galloyl)glucopyranoside or 3-methoxy-5-hydroxyphenol-1-*O*-*β*-D-(6'-*O*-galloyl)glucopyranoside	$C_{20}H_{22}O_{12}$	6.06	negative mode	453.1037	2.16	313.0566, 169.0133, 139.0390, 125.0233
32	Procyanidin B1 or procyanidin B2	$C_{30}H_{26}O_{12}$	6.55	negative mode	577.1349	1.54	425.0876, 451.1039, 407.0768, 289.0718
				positive mode	579.1497	-0.01	409.0913, 163.0388
33	Phyllanthusiin C	$C_{40}H_{30}O_{26}$	6.71	negative mode	925.0955	1.41	615.0638, 605.0789, 309.0245, 300.9989, 275.0197, 247.0245
34	Vanillic acid-4-*O*-(6'-*O*-galloyl)-glucopyranoside	$C_{21}H_{22}O_{13}$	6.98	negative mode	481.0985	1.82	437.1093, 313.0564, 169.0134, 167.0340, 152.0105, 108.0205

35	Ethyl gallate-*O*-hexoside	$C_{15}H_{20}O_{10}$	7.07	negative mode	359.0984	3.01	197.0448, 169.0133,125.0233
36	(–)-Epicatechin	$C_{15}H_{14}O_6$	7.11	negative mode	289.0717	3.65	271.0613, 245.0817, 203.0706, 151.0391, 109.0283
				positive mode	291.0856	-2.35	139.0388, 123.0441
37	5,7-Dihydroxychromone-7-*O*-glucopyranoside	$C_{15}H_{16}O_9$	7.25	negative mode	339.0720	2.75	177.0183
38	2,7,4'-Trihydroxyflavanone-5-*O*-*β*-D-glucopyranoside	$C_{21}H_{22}O_{11}$	7.47	negative mode	449.1089	1.92	287.0560, 269.0455, 259.0609
39*	Emodin	$C_{15}H_{10}O_5$	7.48	positive mode	271.0596	-1.96	243.0646
40	Isomer of 5,7-dihydroxychromone	$C_9H_6O_4$	7.63	negative mode	177.0185	1.44	149.0235, 133.0284, 107.0124, 91.0177
41	12-Hydroxyjasmonic acid glucoside	$C_{18}H_{28}O_9$	7.67	negative mode	387.1660	2.56	207.1021, 163.1118
42	3-Methoxy-4-hydroxyphenol-1-*O*-*β*-D-(6'-*O*-galloyl)glucopyranoside or 2-methoxy-4-hydroxyphenol-1-*O*-*β*-D-(6'-*O*-galloyl)glucopyranoside or 3-methoxy-5-hydroxyphenol-1-*O*-*β*-D-(6'-*O*-galloyl)glucopyranoside	$C_{20}H_{22}O_{12}$	7.70	negative mode	453.1037	2.16	313.0563, 169.0134, 167.0340. 139.0390, 125.0233
43	3(or 3')-O-galloyl(epi)catechin-(4,8')-(epi)catechin	$C_{37}H_{30}O_{16}$	7.81	negative mode	729.1458	1.10	577.1352, 425.0880, 407.0770, 289.0716, 161.0234
44	Trigalloyl glucopyranose	$C_{27}H_{24}O_{18}$	8.12	negative mode	635.0887	1.24	465.0671, 313.0564, 211.0241, 169.0133
				positive mode	637.1032	-0.52	449.0725, 279.0498, 153.0184
45	*p*-Coumaric acid	$C_9H_8O_3$	8.14	negative mode	163.0391	0.55	119.0490
46	Urolithin M5	$C_{13}H_8O_7$	8.15	negative mode	275.0197	4.04	257.0089, 247.0243, 229.0138, 203.0343, 191.0341
47	Trigalloyl glucopyranose	$C_{27}H_{24}O_{18}$	8.48	negative mode	635.0889	1.54	465.0674, 313.0564, 211.0242, 169.0133
				positive mode	637.1034	-0.24	619.0905, 449.0725, 279.0498, 153.0184
48	Benzyl-*O*-*β*-D-glucopyranoside	$C_{13}H_{18}O_6$	8.59	negative mode	269.1027	2.81	/
49	Quercetin-3-*O*-(protocatechuoyl-galloyl)-rhamnoside	$C_{36}H_{32}O_{17}$	8.63	negative mode	735.1563	0.92	583.1091, 447.0930, 301.0348, 243.0295, 125.0232
50	Ethyl gallate	$C_9H_{10}O_5$	8.80	negative mode	197.0448	2.03	169.0133, 125.0233
51	3(or 3')-*O*-galloyl(epi)catechin-(4,8')-(epi)catechin	$C_{37}H_{30}O_{16}$	9.06	negative mode	729.1468	2.44	577.1353, 425.0876, 407.0770, 289.0716, 161.0234, 125.0232
52	Syringic acid	$C_9H_{10}O_5$	9.17	negative mode	197.0449	2.08	182.0212, 166.9976, 153.0547
53	3(or 3')-*O*-galloyl(epi)catechin-(4,8')-(epi)catechin	$C_{37}H_{30}O_{16}$	9.51	negative mode	729.1466	2.19	577.1355, 407.0771, 289.0717, 125.0233
54	*p*-Coumaroyl-1-*O*-galloyl-glucopyranoside	$C_{22}H_{22}O_{12}$	9.75	negative mode	477.1031	0.83	313.0565, 169.0133, 125.0233
55	4-*O*-Methylgallic acid	$C_8H_8O_5$	9.95	negative mode	183.0291	1.53	168.0053, 165.0187, 139.0390, 97.0283
56	Myricetin 3-*O*-hexoside	$C_{21}H_{20}O_{13}$	10.27	negative mode	479.0829	1.76	316.0223, 287.0196, 271.0246
57	5,7-Dihydroxychromone-7-*O*-*β*-D-(6''-*O*-galloyl)-glucopyranoside	$C_{22}H_{20}O_{13}$	10.38	negative mode	491.0827	1.39	313.0565, 177.0184, 125.0232
58	Ethyl brevifolincarboxylate	$C_{15}H_{12}O_8$	10.40	negative mode	319.0457	2.75	183.0291, 165.0184, 153.0183, 139.0390

59	Digalloylarbutin	$C_{26}H_{24}O_{15}$	10.65	negative mode	575.1040	1.48	465.0673, 423.0931, 405.0826, 313.0565, 169.0134, 125.0233
60#	5,7-Dihydroxychromone	$C_{9}H_{6}O_{4}$	10.84	negative mode	177.0185	1.44	149.0235, 133.0284
				positive mode	179.0340	0.64	137.0233
61	Isomer of naringenin 7-*O*-glucopyranoside	$C_{21}H_{22}O_{10}$	10.93	negative mode	433.1137	0.82	343.0821, 313.0717, 271.0611. 193.0135
62	Myricetin-*O*-(*O*-galloyl)-hexoside	$C_{28}H_{24}O_{17}$	11.29	negative mode	631.0936	0.94	479.0828, 317.0301, 271.0245, 178.9977, 151.0026
63#	Ellagic acid	$C_{14}H_{6}O_{8}$	11.33	negative mode	300.9989	3.24	283.9962, 245.0087, 229.0137
				positive mode	303.0132	0.62	257.0075, 201.0180, 173.0231
64	Davidiin	$C_{41}H_{30}O_{26}$	11.36	negative mode	937.0953	1.18	893.1055, 785.0838, 615.0617, 300.9991, 275.0198, 249.0403
				positive mode	939.1103	0.51	303.0130, 277.0338, 153.0182
65	*p*-Coumaroyl-*O*-galloyl-glucopyranoside	$C_{22}H_{22}O_{12}$	11.51	negative mode	477.1036	1.74	313.0565, 169.0134, 125.0233
66	Geraniin/Granatin B	$C_{41}H_{28}O_{27}$	11.67	negative mode	951.0737	0.26	300.9989
				positive mode	953.0877	-1.43	303.0130
67*	Myricitrin	$C_{21}H_{20}O_{12}$	12.10	negative mode	463.0881	2.15	316.0223, 287.0196, 271.0247
				positive mode	465.1022	-1.19	319.0441, 301.0363, 153.0181
68	Myricetin	$C_{15}H_{10}O_{8}$	12.13	positive mode	319.0444	-1.39	290.414, 273.0388, 245.0441, 217.0492, 153.0180
69*	Quercetin-3-*O*-(2''-*O*-galloyl)-*β*-D-glucopyranoside	$C_{28}H_{24}O_{16}$	12.17	negative mode	615.0990	1.53	463.0880, 300.0275
				positive mode	617.1127	-1.69	303.0492, 153.0181
70	(+)-Catechin-3-*O*-gallate or (−)-epicatechin-3-*O*-gallate	$C_{22}H_{18}O_{10}$	12.19	negative mode	441.0827	1.20	289.0717, 169.0133, 125.0232
				positive mode	443.0967	-1.34	291.0857, 153.0180
71	5,5'-Dimethoxyisolariciresinol-*O*-hexoside	$C_{28}H_{38}O_{13}$	12.29	negative mode	581.2236	1.31	419.1710, 404.1474, 389.1241
72	(+)-Catechin-3-*O*-gallate or (−)-epicatechin-3-*O*-gallate	$C_{22}H_{18}O_{10}$	12.37	negative mode	441.0822	1.20	289.0717, 169.0134, 125.0233
				positive mode	443.0974	0.17	291.0861, 273.0756, 153.0183
73	Naringenin-7-*O*-glucopyranoside	$C_{21}H_{22}O_{10}$	12.37	negative mode	433.1135	0.57	343.0821, 313.0716, 271.0609, 193.0134, 119.0490
74#	Quercetin-3-*O*-*β*-D-galactoside	$C_{21}H_{20}O_{12}$	12.64	negative mode	463.0883	2.57	300.0274, 255.0297
				positive mode	465.1027	-0.20	303.0493, 285.0397
75*	Rutin	$C_{27}H_{30}O_{16}$	12.75	negative mode	609.1458	1.21	300.0274, 271.0247, 255.0295, 178.9977, 151.0027
76*#	Quercetin-3-*O*-*β*-D-glucopyranoside	$C_{21}H_{20}O_{12}$	13.00	negative mode	463.0880	2.02	300.0273, 255.0296
				positive mode	465.1028	0.12	303.0494, 285.0396
77	Isolariciresinol-9'-*O*-glucopyranoside	$C_{26}H_{34}O_{11}$	13.20	negative mode	521.2024	1.25	359.1498, 344.1259, 329.1027, 241.0500
78#	Quercetin-3-*O*-glucuronide	$C_{21}H_{18}O_{13}$	13.21	negative mode	477.0670	1.24	301.0352, 255.0293, 178.9977, 151.0026

				positive mode	479.0811	-1,94	303.0494
79	Quercetin-3-*O*-(3''-*O*-galloyl)-*β*-D-glucopyranoside	$C_{28}H_{24}O_{16}$	13.66	negative mode	615.0987	1.02	463.0886, 301.0352, 178.9977, 151.0027,107.0126
80#	Nudiposide	$C_{27}H_{36}O_{12}$	13.68	negative mode	551.2130	1.17	419.1709, 404.1469, 373.1289, 341.1027, 313.0722, 233.0817, 153.0546
81	Geraniin/Granatin B	$C_{41}H_{28}O_{27}$	13.72	negative mode	951.0748	1.42	300.9988
82	Quercetin-3-*O*-arabinoside	$C_{20}H_{18}O_{11}$	14.01	negative mode	433.0773	0.73	300.0274, 271.0247, 255.0295, 227.0334, 151.0026
83	(−)Isolariciresinol-9'-*O*-*β*-D-xylopyranoside or (+)Isolariciresinol-9'-*O*-*β*-D-xylopyranoside	$C_{25}H_{32}O_{10}$	14.13	negative mode	491.1920	1.72	359.1498, 344.1260, 313.1080, 281.0816, 241.0503
84	6'-Acetyl-6(or 3)-feruloylsucrose	$C_{24}H_{32}O_{15}$	14.16	negative mode	559.1665	1.35	499.1458, 337.0927.193.0499, 175.0390, 134.0362
85	Astragalin	$C_{21}H_{20}O_{11}$	14.18	negative mode	447.0930	1.86	285.0385, 284.0326, 255.0296, 227.0344
86	Quercetin-*O*-pentosides	$C_{20}H_{18}O_{11}$	14.37	negative mode	433.0776	1.10	300.0275, 271.0247, 255.0296, 151.0028
87	Quercetin 3-*O*-galloylglucuronide	$C_{28}H_{22}O_{17}$	14.50	negative mode	629.0785	1.85	477.0678, 459.0580, 327.0357, 301.0349, 175.0240, 151.0026
88#	Kaempferol-4-*O*'-rutinoside	$C_{27}H_{30}O_{15}$	14.58	negative mode	593.1511	1.62	285.0400, 255.0297, 227.0345
89	Quercetin-3-*O*-xyloside	$C_{20}H_{18}O_{11}$	14.72	negative mode	433.0773	0.88	300.0274, 271.0247, 255.0296,151.0027
90	3(or 5')-Methoxyisolariciresinol-9'-*O*-xylopyranoside	$C_{26}H_{34}O_{11}$	14.83	negative mode	521.2028	1.25	389.1605, 374.1370, 359.1137, 344.0892
91*	*cis*-*N*-caffeoyltyramine	$C_{17}H_{17}O_4N$	14.85	negative mode	298.1083	3.11	178.0500, 135.0440
				positive mode	300.1229	-2.21	163.0390, 121.0650
92	(−)Isolariciresinol-9'-*O*-*β*-D-xylopyranoside or (+)Isolariciresinol-9'-*O*-*β*-D-xylopyranoside	$C_{25}H_{32}O_{10}$	14.94	negative mode	491.1923	2.21	359.1500, 344.1263, 313.1081, 281.0819, 241.0506
93*#	Quercitrin	$C_{21}H_{20}O_{11}$	15.14	negative mode	447.0929	1.59	300.0274, 271.0247, 255.0296, 151.0026
				positive mode	449.1074	-2.15	303.0492, 287.0544
94	Astragalin 2''-gallate	$C_{28}H_{24}O_{15}$	15.43	negative mode	599.1042	1.84	447.0927,285.0404,257.0452
95	Quercetin-3-*O*-(2''-*O*-protocatechuoyl)-hexoside	$C_{28}H_{24}O_{15}$	15.79	negative mode	599.1034	0.41	463.0869, 301.0352, 271.0249, 255.0295, 178.9977, 151.0027
96	*N*-feruloyloctopamine	$C_{18}H_{19}NO_5$	15.82	negative mode	328.1189	1.23	310.1085, 161.0234, 133.0521
				positive mode	330.1335	-0.45	194.0815, 177.0547, 145.0284, 117.0338
97	Myricetin-3-*O*-(2''-*O*-galloyl)-rhamnopyranoside or myricetin-3-*O*-(3''-*O*-galloyl)-rhamnopyranosid	$C_{28}H_{24}O_{16}$	16.43	negative mode	615.0988	1.23	463.0881, 317.0283, 316.0223, 271.0247, 164.0104, 151.0027
98#	Afzelin	$C_{21}H_{20}O_{10}$	16.60	negative mode	431.0980	1.66	285.0399, 255.0296, 227.0345
				positive mode	433.1134	-0.17	287.0549
99	Isomer of Quercetin-3-*O*-(4''-*O*-acetyl)-α-L-rhamnopyranoside	$C_{23}H_{22}O_{12}$	17.44	negative mode	489.1037	2.00	447.0907, 429.0834, 300.0273, 271.0247, 255.0296, 178.9975, 151.0026
100	Helonioside A	$C_{32}H_{38}O_{17}$	17.53	negative mode	693.2032	1.05	517.1560, 499.1456, 337.0926, 193.0498, 175.0392, 160.0156

101#	*N*-Feruloyltyramine	$C_{18}H_{19}NO_4$	17.58	negative mode	312.1241	1.69	297.1005, 190.0502, 178.0500, 148.0520
				positive mode	314.1389	0.65	194.0810, 177.0546, 145.0284, 117.0337
102	Quercetin-3-*O*-(6''-*O*-trans-feruloyl)-*β*-D-galactopyranoside	$C_{31}H_{28}O_{15}$	17.70	negative mode	639.1352	0.79	463.0868, 301.0349
103	2''-*O*-Galloylquercitrin	$C_{28}H_{24}O_{15}$	17.73	negative mode	599.1036	0.72	447.0916, 301.0351, 151.0026
				positive mode	601.1176	-2.89	413.0876, 303.0490, 153.0180
104	Quercetin-3-*O*-(4''-*O*-acetyl)-α-L-rhamnopyranoside	$C_{23}H_{22}O_{12}$	17.73	negative mode	489.1036	1.57	447.0935, 300.0273, 271.0246, 255.0295, 178.9977, 151.0026
105*	Quercetin	$C_{15}H_{10}O_7$	17.77	negative mode	301.0352	3.13	151.0027, 121.0283, 107.0126
				positive mode	303.0499	-1.91	285.0392, 257.0442, 229.0493
106#	Flazin	$C_{17}H_{12}O_4N_2$	17.80	positive mode	309.0863	-2.11	281.0919, 263.0811, 235.0862, 206.0835, 180.0804
107*	3''-*O*-Galloylquercitrin	$C_{28}H_{24}O_{15}$	17.82	negative mode	599.1039	1.34	447.0940, 301.0349, 271.0247, 255.0296, 178.9977, 151.0027
108#	3,3'-Di-*O*-methylellagic acid	$C_{16}H_{10}O_8$	18.02	negative mode	329.0301	2.85	314.0067, 298.9832, 270.9883
109	Quercetin-3-*O*-(2''-*O*-protocatechuoyl)-rhamnoside	$C_{28}H_{24}O_{14}$	18.33	negative mode	583.1091	1.44	447.0928, 300.0274, 271.0247, 255.0295. 178.9977
110	6'-Acetyl-3(or 6)-feruloyl-6(or 3)-coumaroylsucrose	$C_{33}H_{38}O_{17}$	18.36	negative mode	705.2040	2.15	559.1662, 483.1308, 337.0943, 193.0499, 175.0392, 163.0389,
111	Bistoroside B	$C_{34}H_{40}O_{18}$	18.36	negative mode	735.2141	1.31	559.1661, 337.0931, 193.0498, 175.0392, 160.0156
112	Smilaside A	$C_{36}H_{42}O_{19}$	18.36	negative mode	777.2244	0.92	735.2143, 717.2039, 601.1773, 559.1666, 193.0498, 175.0392, 160.0156
113	3-*O*-Methylquercetin	$C_{16}H_{12}O_7$	18.41	negative mode	315.0510	3.37	300.0274, 271.0247, 255.0296, 243.0296, 148.0155
				positive mode	317.0653	-2.48	302.0417, 274.0466, 153.0181, 137.0233
114	Kaempferol	$C_{15}H_{10}O_6$	19.01	negative mode	285.0402	2.78	255.0299, 239.0345, 227.0342

* Identified by compared with reference standards.

Prototypes of *P. capitatum in* hyperuricemia rat plasma

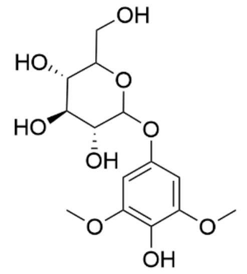

1 or 2

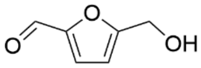

3

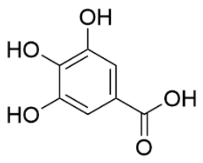

4

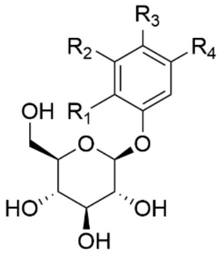

R1	R2	R3	R4	
H	OCH_3	OH	H	Tachioside
OCH_3	H	OH	H	Isotachioside
H	OCH_3	H	OH	Picraquassioside D

5,7 or 16

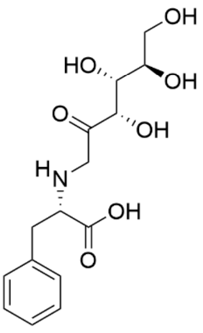

12

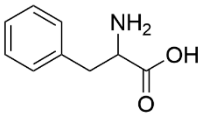

6

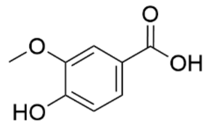

9

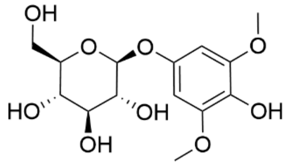

11

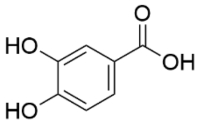

14

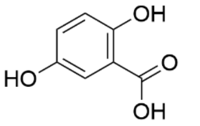

15

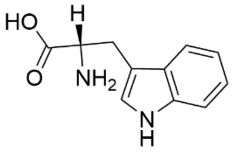

18

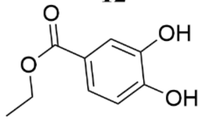

19

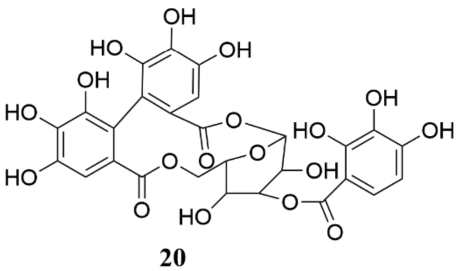

20

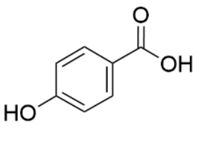

22

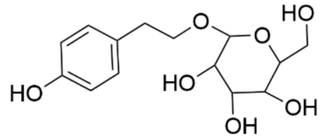

23

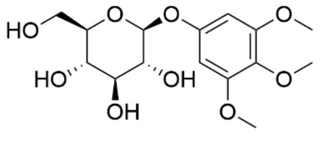

24

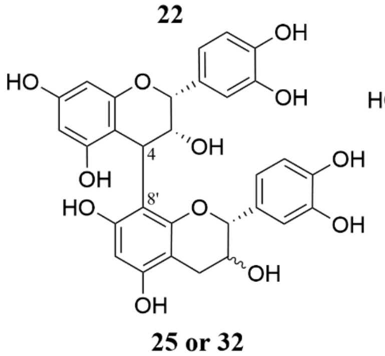

25 or 32

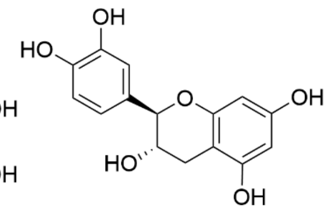

26

Figure S1. The chemical structures of the constituents from *P. capitatum* analyzed by UHPLC-Q-Orbitrap HRMS.

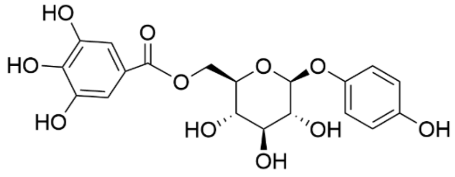

27

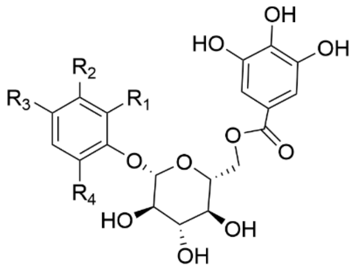

R1	R2	R3	R4
H	OCH3	OH	H
OCH3	H	OH	H
H	OCH3	H	OH

28, 31 or 42

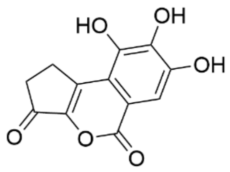

29

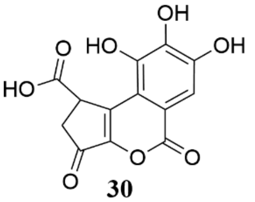

30

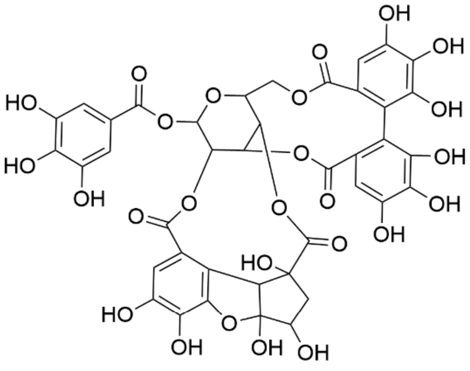

33

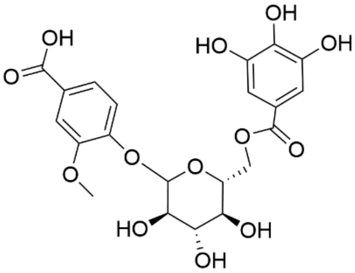

34

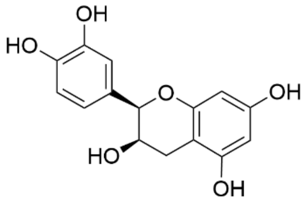

36

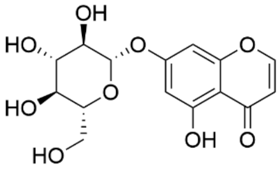

37

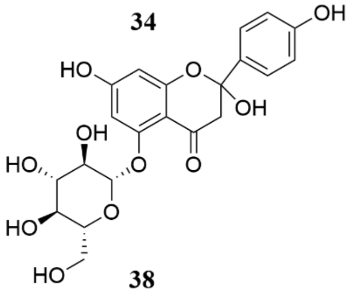

38

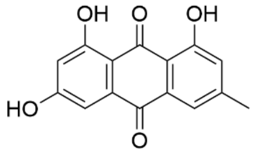

39

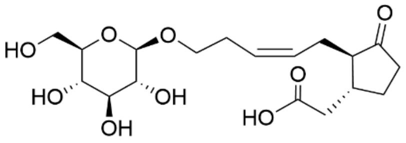

41

Figure S1. The chemical structures of the constituents from *P. capitatum* analyzed by UHPLC-Q-Orbitrap HRMS (*continued*).

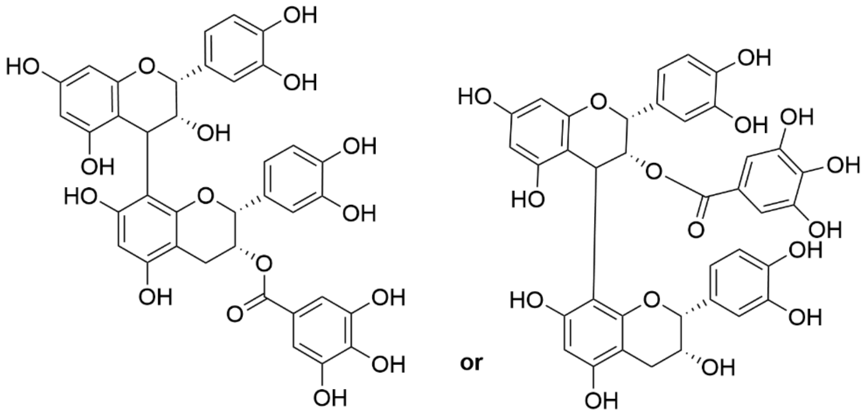

43, 51 or 53

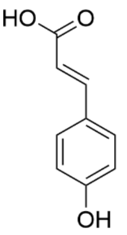

45

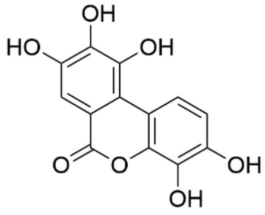

46

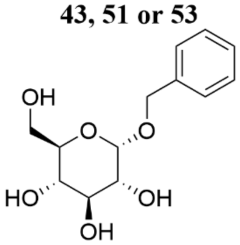

48

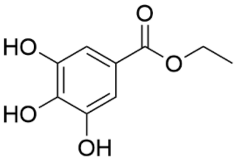

50

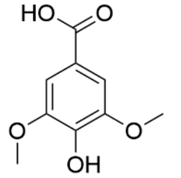

52

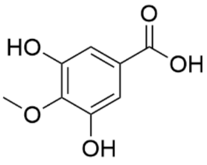

55

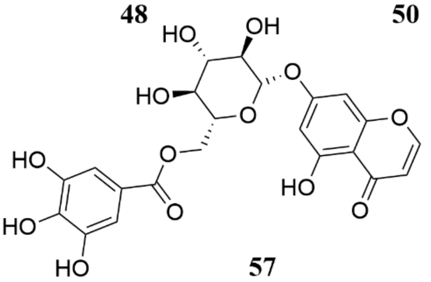

57

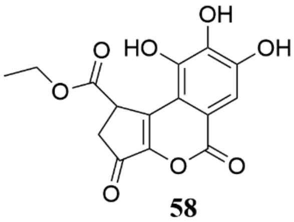

58

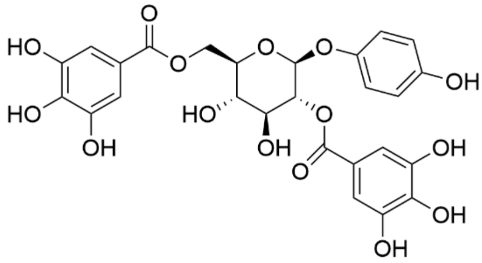

59

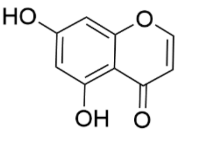

60

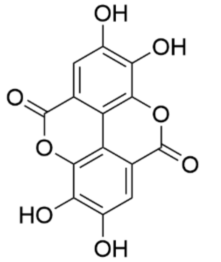

63

Figure S1. The chemical structures of the constituents from *P. capitatum* analyzed by UHPLC-Q-Orbitrap HRMS (*continued*).

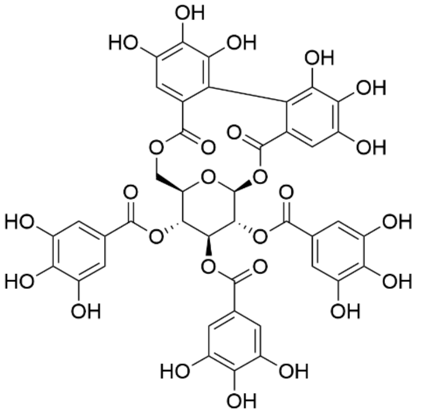

64

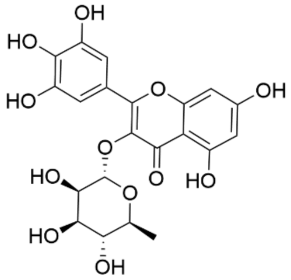

67

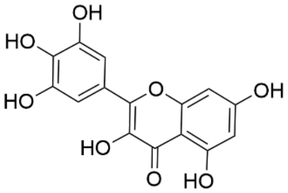

68

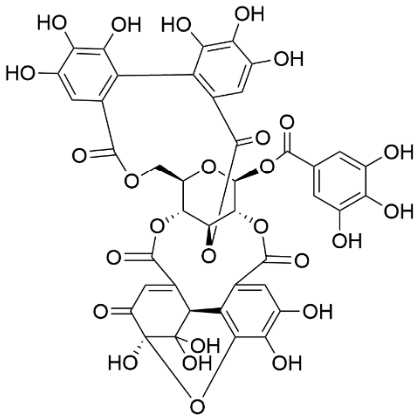

or

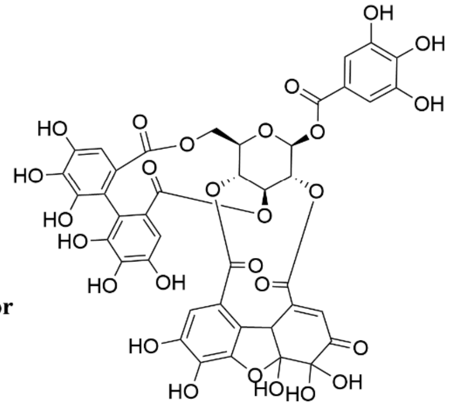

66 or 81

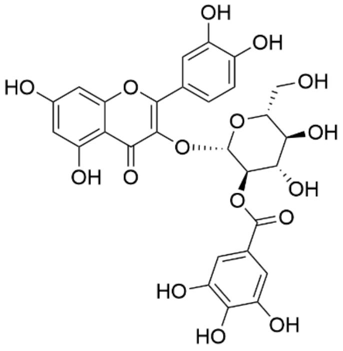

69

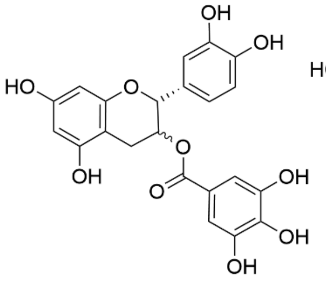

70 or 72

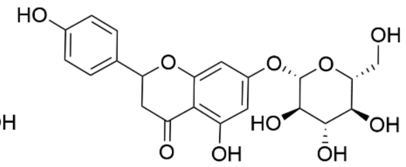

73

Figure S1. The chemical structures of the constituents from *P. capitatum* analyzed by UHPLC-Q-Orbitrap HRMS (*continued*).

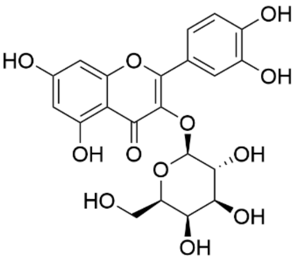

74

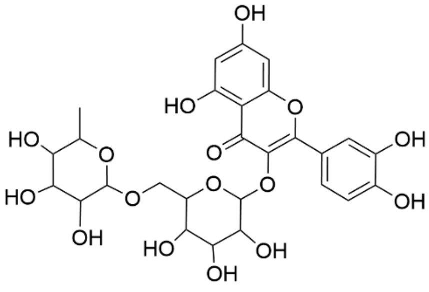

75

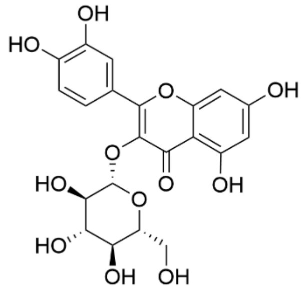

76

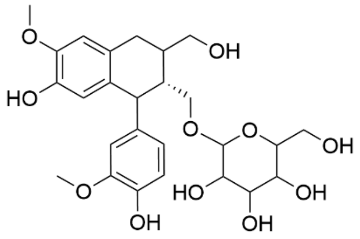

77

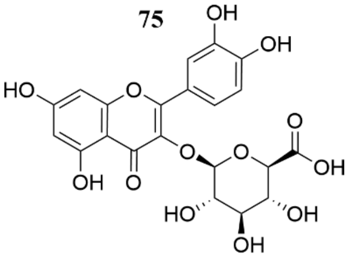

78

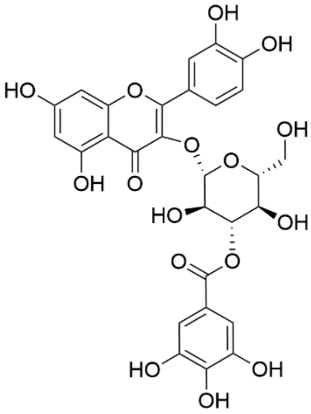

79

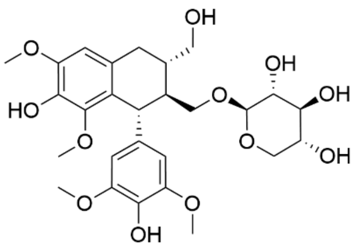

80

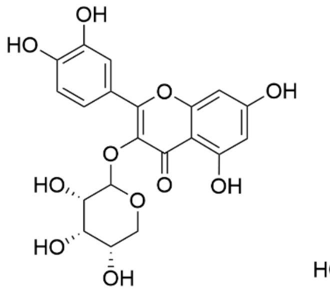

82

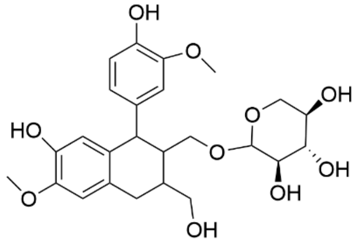

83 or 92

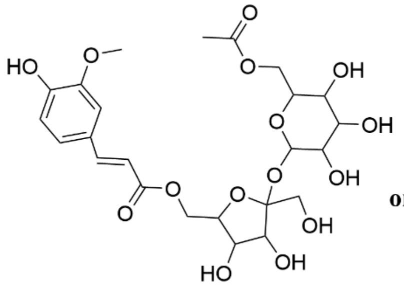

or

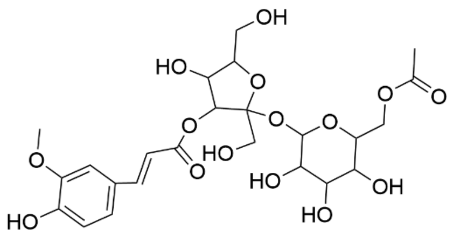

84

Figure S1. The chemical structures of the constituents from *P. capitatum* analyzed by UHPLC-Q-Orbitrap HRMS (*continued*).

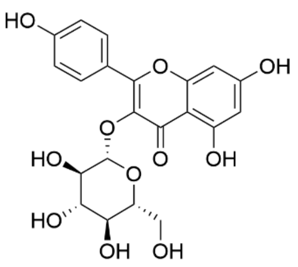

85

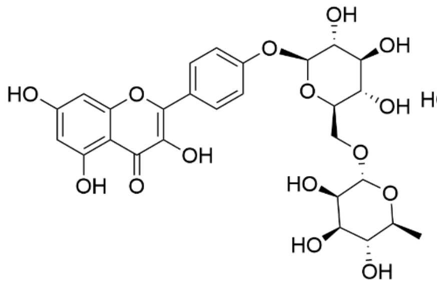

88

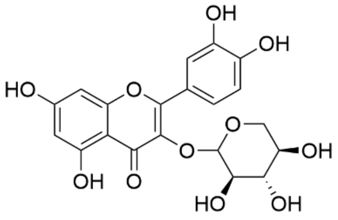

89

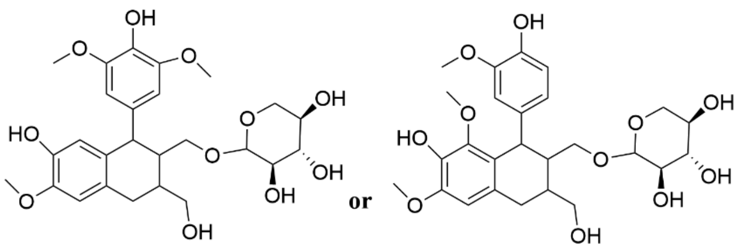

90

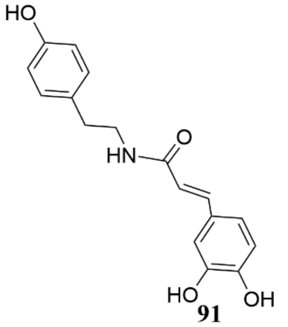

91

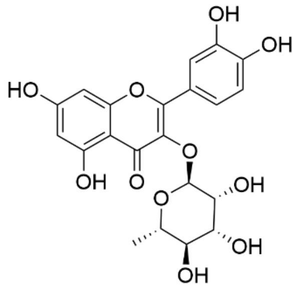

93

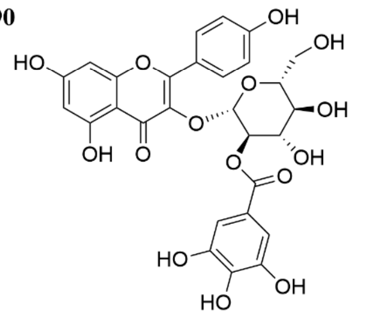

94

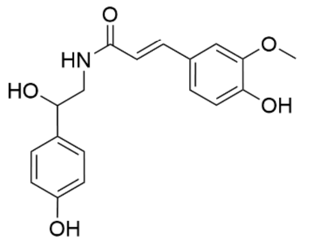

96

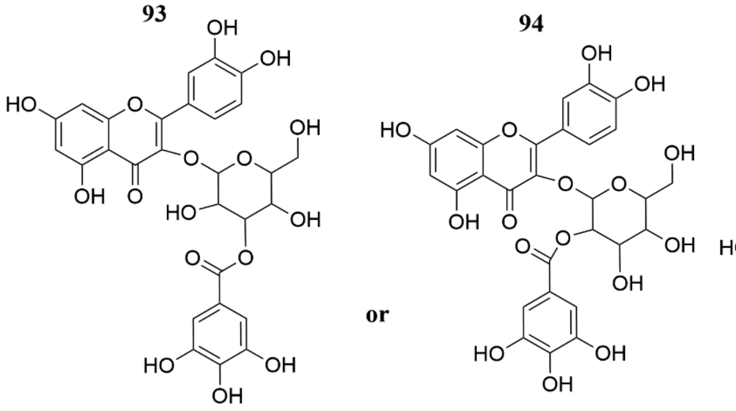

97

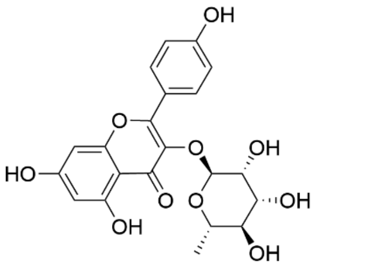

98

Figure S1. The chemical structures of the constituents from *P. capitatum* analyzed by UHPLC-Q-Orbitrap HRMS (*continued*).

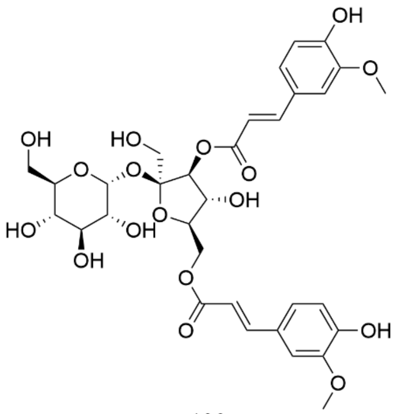

100

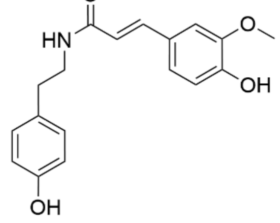

101

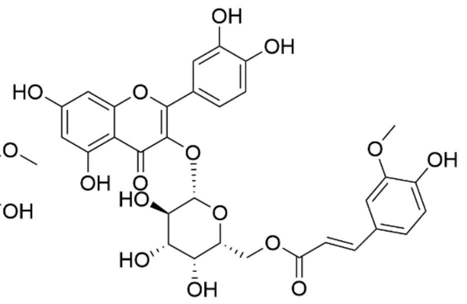

102

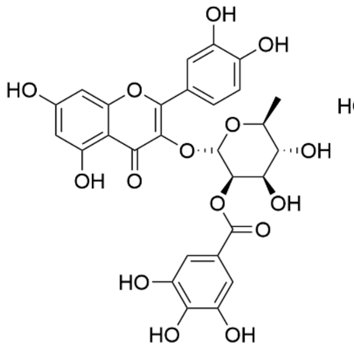

103

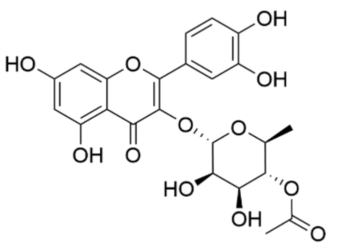

104

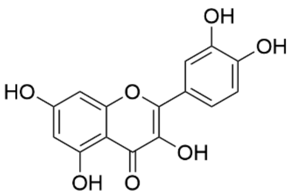

105

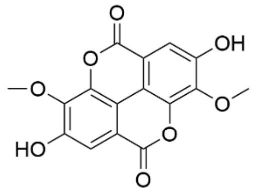

108

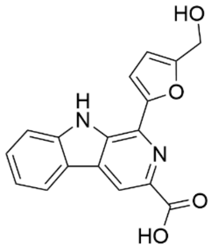

106

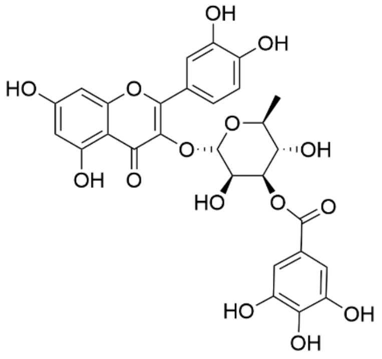

107

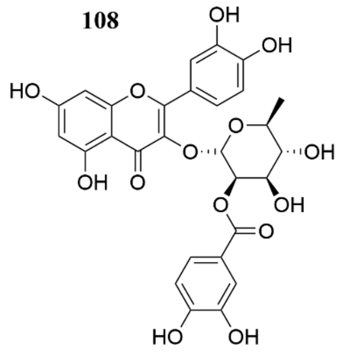

109

Figure S1. The chemical structures of the constituents from *P. capitatum* analyzed by UHPLC-Q-Orbitrap HRMS (*continued*).

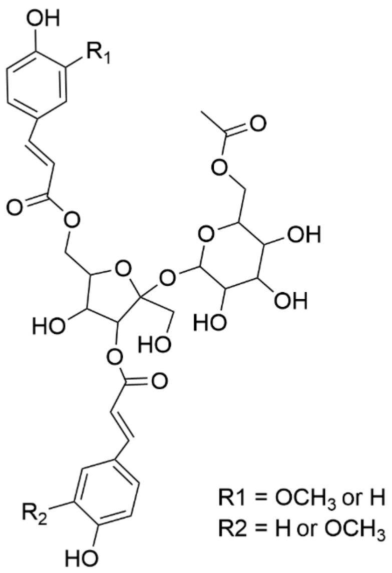

110

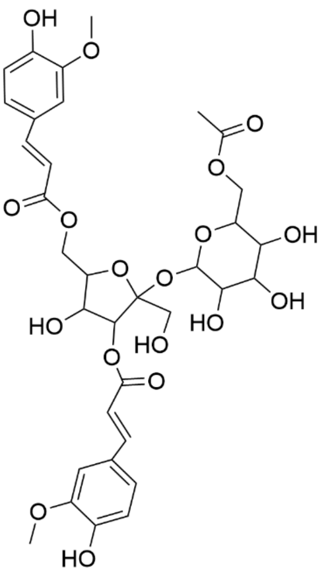

111

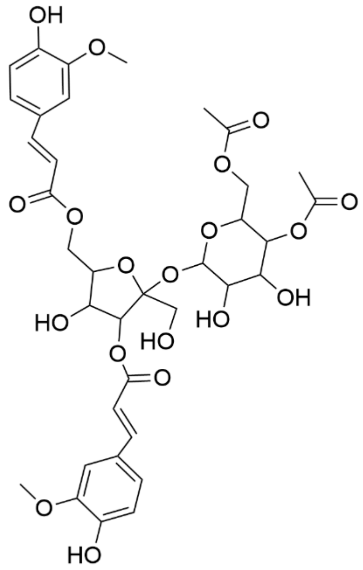

112

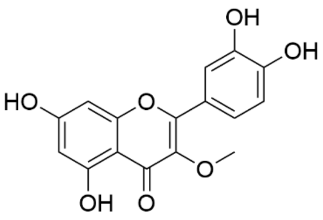

113

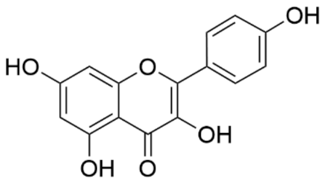

114

Figure S1. The chemical structures of the constituents from *P. capitatum* analyzed by UHPLC-Q-Orbitrap HRMS (*continued*).
